# The Synergistic Effects of the Glutathione Precursor, NAC and First-Line Antibiotics in the Granulomatous Response Against *Mycobacterium tuberculosis*

**DOI:** 10.3389/fimmu.2018.02069

**Published:** 2018-09-12

**Authors:** Garrett Teskey, Ruoqiong Cao, Hicret Islamoglu, Albert Medina, Chaya Prasad, Ramaa Prasad, Airani Sathananthan, Marcel Fraix, Selvakumar Subbian, Li Zhong, Vishwanath Venketaraman

**Affiliations:** ^1^College of life Sciences, Hebei University, Baoding, China; ^2^Graduate College of Biomedical Sciences, Western University of Health Sciences, Pomona, CA, United States; ^3^Department of Basic Medical Sciences, College of Osteopathic Medicine of the Pacific, Western University of Health Sciences, Pomona, CA, United States; ^4^Department of Biological Sciences, California State Polytechnic University, Pomona, CA, United States; ^5^Department of Internal Medicine, College of Osteopathic Medicine of the Pacific, Western University of Health Sciences, Pomona, CA, United States; ^6^Department of Clinical Sciences, College of Osteopathic Medicine of the Pacific, Western University of Health Sciences, Pomona, CA, United States; ^7^Public Health Research Institute (PHRI), New Jersey Medical School, Rutgers University, Newark, NJ, United States

**Keywords:** tuberculosis, cytokines, antibiotics, antitubercular, *Mycobacterium tuberculosis*, type 2 diabetes

## Abstract

*Mycobacterium tuberculosis* (*M. tb*), the causative bacterial agent responsible for tuberculosis (TB) continues to afflict millions of people worldwide. Although the human immune system plays a critical role in containing *M. tb* infection, elimination proves immensely more challenging. Consequently, there has been a worldwide effort to eradicate, and limit the spread of *M. tb* through the conventional use of first-line antibiotics. Unfortunately, with the emergence of drug resistant and multi-drug resistant strains of *M. tb* the archetypical antibiotics no longer provide the same ascendancy as they once did. Furthermore, when administered, these first-line antibiotics commonly present severe complications and side effects. The biological antioxidant glutathione (GSH) however, has been demonstrated to have a profound mycobactericidal effect with no reported adverse consequences. Therefore, we examined if N-Acetyl Cysteine (NAC), the molecular precursor to GSH, when supplemented in combination with suboptimal levels of standalone first-line antibiotics would be sufficient to completely clear *M. tb* infection within *in vitro* derived granulomas from healthy subjects and individuals with type 2 diabetes (T2DM). Our results revealed that by virtue of immune modulation, the addition of NAC to subprime levels of isoniazid (INH) and rifampicin (RIF) was indeed capable of inducing complete clearance of *M. tb* among healthy individuals.

## Introduction

*Mycobacterium tuberculosis* (*M. tb*), the infectious pathogen responsible for tuberculosis (TB), caused 10.4 million new cases of active disease and 1.8 million deaths worldwide in 2016 alone (1). Additionally, it is estimated that currently one third of the world's population is latently infected with *M. tb* (1). *M. tb*'s mode of transmission is mitigated via aerosol droplets typically by means of coughing, which if inhaled leads to bacterial seeding within the host's lower respiratory tract. Although TB primarily affects the lung parenchyma, it may also impact bone, the central nervous system, and other organ systems (2). Upon infection, a competent immune system governs the integral processes of host defense against the contagion (3, 4).

Archetypically, early innate defenses fail to completely eradicate the invading *M. tb* (5). Consequently, macrophages commit to the cytokine mediated recruitment of additional immune cells, which form an early infectious lesion and beginnings of a granuloma (6, 7). Upon suitable cell signaling, monocytes in the proximity of the infectious region will differentiate into macrophages, a crucial step in granuloma formation and *M. tb* containment (8). A granuloma is a host defense mechanism orchestrated by various immune cells including: macrophages, dendritic cells, T cells, fibroblasts, epithelioid histiocytes, giant cells, and natural killer cells to control and contain a *M. tb* infection by encapsulating the bacteria in order to limit its spread and obstruct it from nutriment; thus, rendering it latent. This granulomatous containment of *M. tb* within the lungs is commonly referred as latent tuberculosis (LTBI), as an individual is no longer infectious at this stage. However, if an individual with LTBI becomes immunodeficient or immunocompromised the granuloma can undergo liquefaction and the latent bacteria undergo reactivation to cause an active TB disease (9).

Individuals with Type 2 diabetes mellitus (T2DM) are typically more vulnerable to bacterial infections, especially *M. tb* as a result of compromised cell mediated immunity (10, 11). T2DM is characterized by hyperglycemia caused by insulin resistance, and accounts for 90–95% of the total prevalence of diabetes (12). Glycated hemoglobin (HbA1c) is indicative of the levels of plasma glucose over a period of several weeks and is correspondingly used as a diagnostic for T2DM. Roughly 10% of TB cases are linked to diabetes, and it is estimated that patients with T2DM are nearly three times more likely to develop an active TB compared to healthy individuals. Conjointly, patients with both T2DM and TB habitually have worse outcomes, thus the risk of mortality is approximately double for those with the comorbidity (13, 14).

Although *M. tb* proliferates within alveolar macrophages, its presence within an adept immune system will induce the release of beneficial cytokines, which in turn activate these macrophages to stimulate their mycobactericidal faculties (15). The cytokines released by macrophages and the other aforementioned cell types possess essential regulatory effects and participate in the host defense against *M. tb* throughout the formation of the granuloma and the subsequent containment and extermination of the mycobacteria (15, 16). The interaction between *M. tb* and granulomatous cells of both the innate and adaptive immune system result in the constant secretion of cytokines; most notably, tumor necrosis factor-α (TNF-α), interleukin-10 (IL-10), IL-6, IL-2, IL-12, and interferon gamma (IFN-γ) (15–17). Additionally, the functionality of polarized CD4+ T cells are largely based on these cytokine secretions. Type-1 T helper (Th1) cells produce IFN-γ, IL-2, and TNF-α, a mechanism thought to be a pivotal in the suppression of *M. tb* growth and replication (18). IFN-γ stimulates dendritic and macrophage differentiation and activation, as well as cell-mediated immunity in the interest of control over intracellular infections (19). TNF-α is predominantly responsible for the cellular recruitment and aggregation necessary for granuloma formation as well as the production of other cytokines, such as IL-1, IL-6, and the indirect stimulation of IFN-γ and IL-2, which can then amplify the TNF-α production (20–23). These cytokines serve in the immunomodulatory role that helps establish granuloma formation, macrophage differentiation, and mediate mycobactericidal potentiality in conjunction with their inflammatory functionality.

The Center for Disease Control and Prevention (CDC) recommends that the preferred treatment regimen for active TB includes the four first-line antibiotics: Isoniazid (INH), Rifampin (RIF), Ethambutol (EMB), and Pyrazinamide (PZA) (12). However, there are a myriad severe complications and side effects which can come from consuming these aforementioned antibiotics. For example, patients taking INH often experience elevated levels of liver enzymes, supplementary to a black box warning for severe and sometimes fatal hepatitis (24, 25). Individuals taking RIF are known to experience: fever, gastrointestinal disturbances, rashes, and immunological reactions. More severe side effects, such as peripheral neuropathy, joint pain, and visual impairment, are also possible when these antibiotics are administered (24–26). However, the most adverse reaction a patient may experience is hepatotoxicity; therefore, patients often undergo frequent liver functionality tests to detect early liver damage (24, 25).

To investigate a novel therapeutic agent to augment the treatment of TB we examined the prophylactic effects of N-acetyl cysteine (NAC), the precursor molecule to the synthesis of the biologic antioxidant glutathione (GSH), when supplemented with individual first-line antibiotics. GSH is a tripeptide comprised of glutamate, cysteine and glycine, which primarily functions in the protection of cells and tissues by reducing oxidative damage thus establishing redox homeostasis within the body (27). Our laboratory has previously demonstrated that GSH has direct antimycobacterial effects (28–30). The primary mechanism of NAC/GSH anti-mycobacterial effects could be due to a shift in redox balance. Mycobacteria lack GSH and possess an alternative thiol, mycothiol to regulate redox homeostasis. Therefore, presence of millimolar concentration of GSH (physiological concentrations) inside the macrophages can cause reductive stress in mycobacteria leading to inhibition in the growth of *M. tb* (28–30). Both GSH and NAC have been previously shown to diminish TB pathology and inflammation by immune-modulation, as well as possess antimycobacterial potentiality (31–35). GSH and NAC's potent anti-inflammatory effects are thought to be by means of inhibiting the activation of nuclear factor-κB (NF-κB) as well as the specific inhibition of other proinflammatory cytokine synthesis (36–38). In both experimental animal models as well as clinical studies, NAC has been shown to have a protective effect against liver damage from anti-tuberculosis medications (39–42). Additionally, Amaral et al. reported that NAC exhibits potent anti-mycobacterial effects and may limit *M. tb* infection and disease both through suppression of the host oxidative response and through direct antimicrobial activity (35). Furthermore, Vilcheze et al. recently demonstrated that the combination of cysteine or other small thiols with either isoniazid or rifampicin prevents the formation of drug-tolerant and drug-resistant *M. tb* cultures by shifting the menaquinol/menaquinone balance toward a reduced state which stimulates *M. tb* respiration and converts persister cells to metabolically active cells. This prevention of both persister cell formation and drug resistance ultimately leads to mycobacterial cell death (43).

Therefore, using *in vitro* generated granulomas from both healthy and T2DM individuals, we tested the synergistic effects of NAC (GSH) as an immunoadjuvant administered in supplementation with either INH, RIF, or EMB in opposition to the highly virulent Erdman strain of *M. tb*. Our findings indicate that there was not only a significant reduction in the intracellular viability of *M. tb*, but an increase in beneficial immunomodulatory effects was also observed when the first-line antibiotics INH, RIF, or EMB were supplemented with NAC, in comparison to the treatment of the standalone antibiotics.

## Materials and methods

### Statement of ethics

This study was approved by both the Institutional Review Board (IRB) and the Institutional Biosafety Committee (IBC) of the Western University of Health Sciences. All study participants were above the legal age of consent at the time of participation and written informed consent was obtained from the volunteers prior to their participation in the study.

### Subject recruitment

Thirteen subjects (8 healthy individuals and 5 participants with T2DM) were recruited for this study. Participants belonging to the healthy group were in the age group between 20 and 65 years and had no history of HIV infection or TB. Additionally, the healthy individuals presented a glycated hemoglobin (HbA1c) of less than 5.7%. Individuals with T2DM who participated in this study were recruited without any preference for age, gender, or ethnicity. The inclusion criteria for T2DM group required that the participants have documentation of a T2DM diagnosis through Dr. Airani Sathananthan at the Patient Care Center of Western University of Health Sciences, and a recorded HbA1c above 8%. Exclusion criteria for the T2DM patients stipulated that they presented no history of autoimmune disease, HIV infection, hepatitis, or TB and were not currently being treated with metformin, as previous studies have indicated that metformin has protective effects against *M. tb* (44, 45). After obtaining signed informed consent, forty milliliters (mL) of blood was drawn for research laboratory tests from the volunteers.

### Preparation of bacteria for infection assays

An Erdman strain of *M. tb* was used for all our infection studies, which expresses green fluorescent protein (GFP). This Erdman strain of *M. tb* (henceforth referred to as *M. tb*) was provided by Dr. Selvakumar Subbian at Rutgers New Jersey Medical School. Erdman strain of *M. tb* has slightly faster doubling time and therefore is considered more virulent compared to the standard laboratory H37Rv strain (46). *M. tb* was cultured in 7H9 media medium (Hi Media, Santa Maria, CA, USA) supplemented with albumin dextrose complex (ADC) (GEMINI, USA) and incubated at 37°C until the bacteria reached logarithmic phase of growth. Bacterial cultures were processed for infection once the static cultures reached peak logarithmic growth phase (indicated by an optical density between 0.5 and 0.8 at A600). The processing steps involve washing *M. tb* cultures with PBS (Sigma, St Louis, MO, USA), and subsequently disaggregating the bacterial clumps by vortexing five times with 3 mm sterile glass beads at 3 min intervals. *M. tb* suspension was then filtered using a 5 μm syringe filter to remove any remaining bacterial aggregations. The single cell suspension of now processed *M. tb* was then serially diluted and plated on 7H11 agar (Hi Media, Santa Maria, CA, USA) to determine the bacterial numbers present in the processed stock. Aliquots of processed bacterial stocks were then frozen and stored in cryogenic freezer at −80°C. At the time of the experimental trial, the frozen-processed stocks of *M. tb* were thawed and used for the infection. All infection studies and handling of the *M. tb* was done inside a certified biosafety level 3 facility (BSL-3).

### To determine the direct antimycobacterial effects of NAC and synergistic effects of NAC added in conjunction with an antibiotic

To determine the direct effects of NAC and synergistic effects of NAC+antibiotic in altering the survival of *M. tb*, bacteria (6 × 10^4^ /well) were grown in 7H9 media in 24 well tissue culture plates (Corning, NY, USA) in the presence and absence of NAC and NAC+antibiotic for 15 days. *M. tb* cultures were then either sham-treated (control) or treated with the minimum inhibitory concentration (MIC) of various first-line antibiotics with or without NAC supplementation, comprising of: INH (0.125 micrograms/ml), INH (0.125 micrograms/ml) + NAC (10 mM), RIF (0.125 micrograms/ml), RIF (0.125 micrograms/ml) + NAC (10 mM), EMB (8.0 micrograms/ml) or EMB (8.0 micrograms/ml) + NAC (10 mM). With the exception of control category, all treatment groups received their preexisting additives every 4 days with the same aforestated concentration until their termination. *M. tb* cultures were maintained at 37°C, with 5% CO_2_ until they were terminated at 8 and 15 days to determine the viability of *M. tb*. *M. tb* viability was ascertained by plating the diluted samples on 7H11 agar medium (Hi Media, Santa Maria, CA, USA) enriched with albumin dextrose complex (ADC) (Gemini, USA).

### Isolating peripheral blood mononuclear cells from human blood

Peripheral blood mononuclear cells (PBMC) were isolated from the whole blood of both healthy individuals and T2DM patients by density gradient centrifugation using ficoll histopaque (Sigma, St Louis, MO, USA), a high density-pH neutral polysaccharide solution. PBMCs were then suspended in RPMI (Sigma, St Louis, MO, USA) containing 5% human AB Serum (Sigma, St Louis, MO, USA) and distributed at 6 × 10^5^ cells/well into 0.001% poly-lysine (Sigma, St Louis, MO, USA) coated 24-well plates (Corning, NY, USA) for further studies.

### PBMC infection and treatment of granulomas

PBMCs were infected with processed virulent *M. tb* at a multiplicity of infection (MOI) of 0.1:1 cell ratio (approximately 6 × 10^4^ bacteria were added to 6 × 10^5^ PBMCs). Infected PBMCs were then either sham-treated or treated with the minimum inhibitory concentration (MIC) of various first-line antibiotics with or without NAC supplementation, comprising of: INH (0.125 micrograms/ml), INH (0.125 micrograms/ml) + NAC (10 mM), RIF (0.125 micrograms/ml), RIF (0.125 micrograms/ml) + NAC (10 mM), EMB (8.0 micrograms/ml) or EMB (8.0 micrograms/ml) + NAC (10 mM). With the exception of control category, all treatment groups received their preexisting additives every 4 days with the same aforestated concentration until their termination. Infected PBMCs were maintained at 37°C, with 5% CO_2_ until they were terminated at 8 and 15 days post-infection, the time determined for sufficient granuloma formation.

### Termination of granulomas to determine the survival of *M. tb*

Previous research by Kapoor et al. demonstrated that granuloma formation occurs approximately seven days post PBMC-infection with *M. tb* (47). Therefore, we terminated and harvested our *in vitro* granulomas at 8 and 15 days post infection to determine the intracellular survival of *M. tb* inside the untreated, antibiotic-treated and antibiotic + NAC-treated granulomas. During termination, cell free supernatants from each well were collected and stored, and granulomas were harvested by adding 250 μl of ice-cold, sterile 1X PBS (Sigma, St, Louis, MO, USA) followed by gentle scraping to achieve maximum recovery of granuloma lysates from the wells. Lysates were then efficaciously vortexed followed by a freeze/thaw cycle in order to ensure that there is sufficient rupture of cells and release of intracellular *M. tb*. The collected lysates and supernatants were then diluted as necessary in sterile 1X PBS (Sigma, St, Louis, MO, USA) and plated on 7H11 agar medium (Hi Media, Santa Maria, CA, USA) enriched with ADC (GEMINI, USA) and incubated at 37°C for 3 weeks, in order to evaluate the mycobacterial growth or survival under different treatment conditions by counting the colony forming units (CFUs).

### Cytokines measurements of granuloma culture medium

Levels of cytokines such as IFN-γ, TNF-α, IL-12, IL-10, and IL-6, were measured in the granuloma supernatants at 8 and 15 days post-infection to determine the effects of antibiotic and antibiotic + NAC treatments in altering the production of cytokines. Cytokine levels were measured by enzyme-linked immunosorbent assay (ELISA). The assay was performed as per the manufacturer's protocol (Affymetrix, San Diego, CA, USA).

### Assessment of GSH levels in the cellular lysates

The effects of antibiotic and antibiotic + NAC treatments in altering the levels of GSH in *M. tb*-granulomas was determined by measuring the levels of GSH in the granuloma lysates by colorimetric method using an assay kit from Arbor Assay (K006-H1). Granuloma lysates were first comprehensively mixed with an equal volume of cold 5% sulfosalicylic acid (SSA), followed by incubation for 10 min at 4°C, and subsequently centrifuged at 14,000 rpm for 10 min. The GSH was thereupon measured in the supernatants following the manufacturer's instructions. The rGSH (reduced GSH) was then calculated by subtracting GSSG (oxidized glutathione) from the total GSH. All measurements were accordingly normalized by the total protein levels and the results were reported in moles of GSH per gram of protein.

### Quantifying levels of malondialdehyde

Malondialdehyde (MDA), a byproduct of lipid peroxidation serves as an important marker for oxidative stress. MDA levels were measured calorimetrically using an assay kit (TBARS assay kit) from Cayman Chemicals. The assay principle is based on reaction between MDA and thiobarbituric acid at 100°C leading to a color change, which can be measured calorimetrically at 535 nm. All measurements were corrected for total protein levels.

### Staining and imaging of granulomas

The experimental setup in each trial includes setting aside granulomas for microscopic studies (light and fluorescent). This was accomplished by generating granulomas on cover glass positioned in wells of tissue culture plates. The granulomas on cover glasses were dedicated for cellular imaging. Granulomas terminated at 15 days post-infection were fixed with 4% paraformaldehyde (PFA) for 1 h at room temperature and then washed with 1X PBS for 5 min to remove any cell debris. Fixed granulomas on the cover glasses were then stained with Hematoxylin and Eosin (H&E) (Poly Scientific, Bay Shore, NY, USA) for 2 min at room temperature and destained with tap water. The coverglass with the stained granulomas were mounted onto glass slides with mounting media (HistoChoice).

The alternative cover glasses with granulomas were used to determine the extent of acidification of the *M. tb*-containing phagosomes within the macrophages present.in the granulomatous. LysoTracker red DND99 (Introgen, Eugene, Oregon, USA), an acidotropic dye and a weak base conjugated to a red fluorophore, was added to the cells on the designated coverglass wells on the day of infection. LysoTracker freely permeates the cell compartment and get trapped inside the acidified compartments such as lysosomes. Acidified compartments were therefore labeled with LysoTracker. Since the *in vitro* granulomas were generated using *M. tb* that express GFP, LysoTracker staining will help determine the whether or not the pathogen is inside the acidified compartment inside the cells. Granulomas terminated at 15 days post-infection were fixed with 4% PFA and cover glasses were mounted on glass slides with 4.5 μL of mounting media containing DAPI (Vector Laboratories, Burlingame, CA, USA). The stained slides were observed under an inverted fluorescent microscope to quantify the extent of co-localization of GFP-expressing *M. tb* with LysoTracker. Images were obtained using an integrated digital camera and analyzed by counting the green, red and yellow bacterial cells. Fixed granulomas on cover glasses were also stained for necrosis, reactive oxygen intermediate (ROI) production, and for the expressions of CD86 (marker on macrophages and dendritic cells), CD4 and CD8. ROI production in the granulomas was determined by cellROX staining. Untreated and NAC-treated granulomas from healthy subjects and individuals with T2DM were treated with 5 μM cellROX green reagent (Life Technologies, C10444) and incubated at room temperature for 30 min in dark. Stained slides were observed under an inverted fluorescent microscope to evaluate the extent of ROI production. Untreated and NAC-treated granulomas from healthy subjects and individuals with T2DM were also treated with 0.1 μg/ml of Propidium Iodide (eBioscience) and incubated at room temperature for 10 min in dark. The stained coverglasses were mounted on glass slides and observed under an inverted fluorescent microscope to determine the extent of cell death due to necrosis. To determine the types of immune cells, present in the *in vitro* granulomas, fixed samples from healthy subjects and individuals with T2DM were stained with antibodies conjugated to the fluorescent markers such as CD86-PE (eBioscience, 12–0149), CD4-Cy5 (eBioscience, 15–0049), and CD8-Cy5 (eBioscience, 15–0088), and incubated in the dark for 30 min. Slides were observed under an inverted fluorescent microscope to determine the cell types present in the granulomas.

### Statistical analysis

Statistical data analysis was performed using GraphPad Prism Software version 7. Levels of cytokines, GSH, MDA and CFUs were compared between untreated control, antibiotic-treatment and treatment with antibiotics in conjunction with NAC using the unpaired *t*-test with Welch correction. Reported values are in means. *p* < 0.05 was considered significant (^*^p < 0.05, ^**^p < 0.005).

## Results

### Survival of erdman strain of *M. tb* in growth media

**Figure 1:**
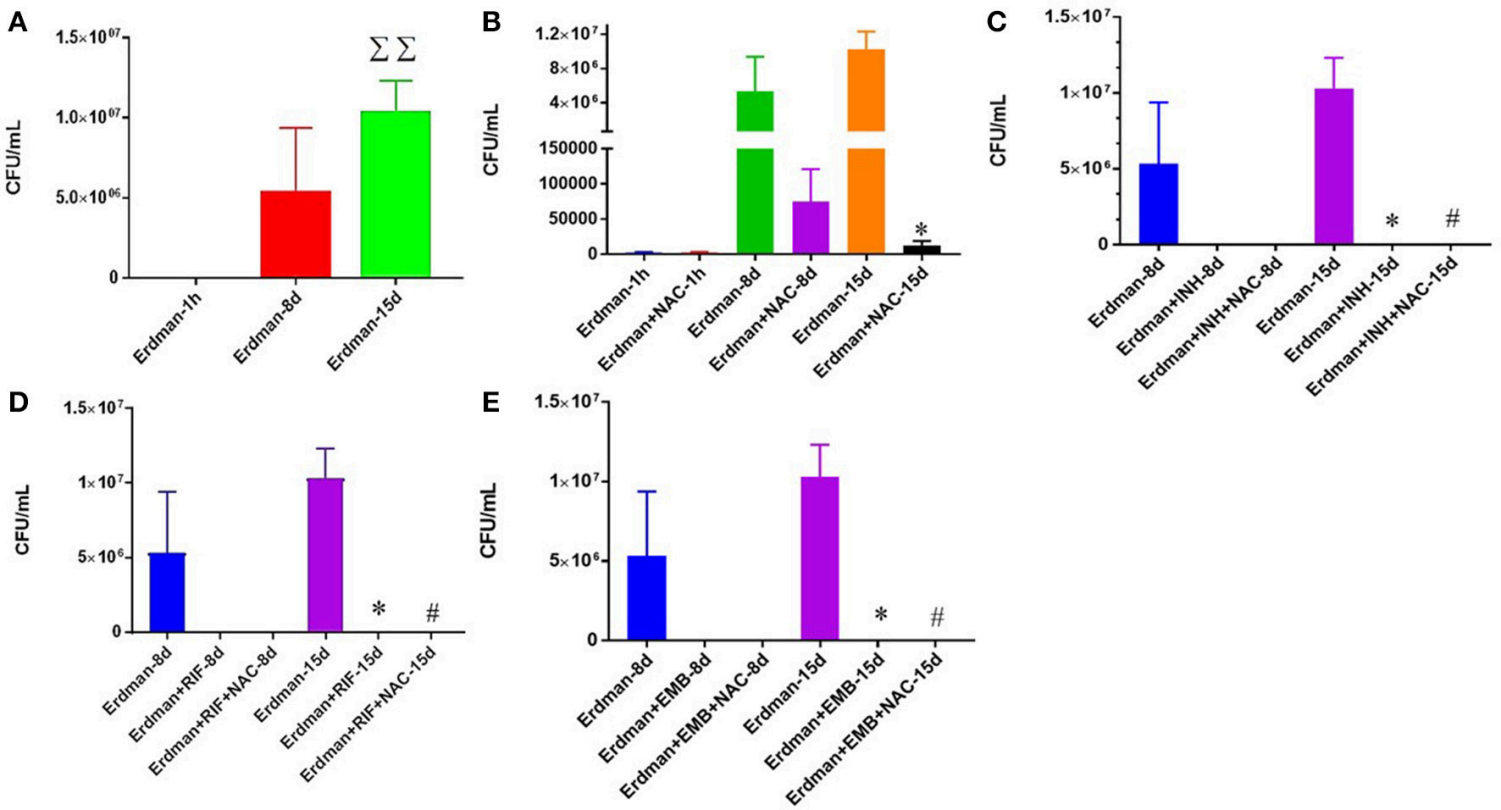
Survival of Erdman strain of *M. tb* in 7H9 media. *M. tb* grown in 7H9 media containing no additives **(A)**, *M. tb* grown in 7H9 media containing NAC **(B)**, *M. tb* grown in 7H9 media containing INH and INH+ NAC **(C)**, *M. tb* grown in 7H9 media containing RIF and RIF + NAC **(D)**, and *M. tb* grown in 7H9 media containing EMB and EMB+ NAC **(E)**. There was a significant increase in bacterial numbers at 15 days when *M. tb* was grown in 7H9 in the absence of any additives **(A)**. There was a significant reduction in the bacterial numbers at 15 days when *M. tb* was grown in 7H9 in the presence of NAC **(B)**, INH and INH+NAC **(C)**, RIF and RIF+NAC **(D)** and EMB and EMB+NAC **(E)**. Data represent means ±SE from experiments performed in triplicate. ^*^p < 0.05 when comparing samples to their respective controls. #*p* < 0.05 when comparing samples to their respective antibiotic only treatments. ΣΣ*p* < 0.005 when comparing Erdman only samples at 15 days to Erdman only samples at 1 h.

We first tested the direct effects of NAC and synergistic NAC + antibiotic combination in altering the growth of *M. tb* grown in 7H9 media. Approximately, 6 × 10^4^ *M. tb* were maintained in 7H9 media in 24-well plates and incubated for 15 days in the presence and absence of additives. *M. tb* cultures were terminated at 8-day and 15-day time points. In sham-treated control group, there was a significant increase in the bacterial survival at the 15-day time point (Figure 1A). To identify whether NAC has direct killing effects on *M. tb*, we treated the Erdman strain with 10 mM NAC four times during the study, and this resulted in a significant inhibition in the growth of *M. tb* at both 8 and 15 days time points (Figure 1B). There was a significant decrease at 15 days (Figure 1B). The synergistic effects of NAC with front line antibiotics against *M. tb* was also tested in the absence of host cells. The trends for all the 3 antibiotics, INH, RIF and EMB, were the same, i.e. there was a statistically significant decrease in the viability of *M. tb i*n single antibiotic categories (Figures 1C–E). Administration with the combination of antibiotics and NAC had a completely clearance of bacteria (Figures 1C–E).

### *M. tb* survival and effector responses against the pathogen inside NAC-treated granulomas from healthy subjects

**Figure 2:**
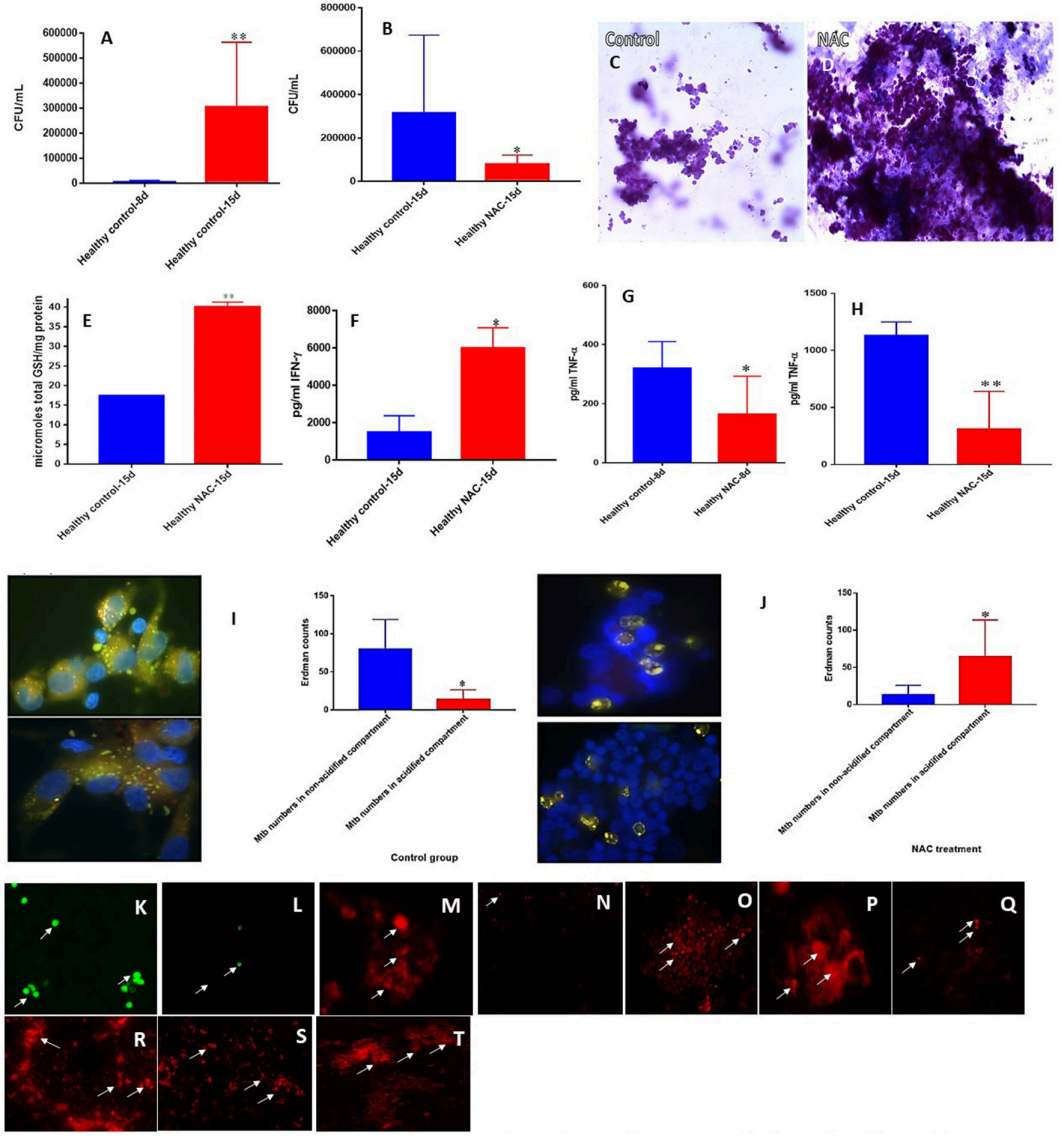
NAC effects on *in vitro* granulomas developed using immune cells from healthy subjects. Survival of *M. tb* inside untreated granulomas **(A)**, Survival of *M. tb* inside NAC-treated granulomas **(B)**, Hematoxylin and Eosin staining of untreated **(C)** and NAC-treated granulomas **(D)** from healthy individuals, determination of GSH levels at 15 days post-infection **(E)**, IFN-γ levels at 15 days post-infection **(F)**, TNF-α levels at 8 days **(G)** and 15 days **(H)** post-infection, phagosome acidification at 15 days post-infection **(I,J)**, cellROX staining of untreated **(K)** and NAC-treated granulomas **(L)** from healthy individuals. Propidium iodide staining of untreated **(M)** and NAC-treated granulomas **(N)** from healthy individuals. CD4 staining of untreated **(O)** and NAC-treated granulomas **(P)**. CD8 staining of untreated **(Q)** and NAC-treated granulomas **(R)**. CD86 staining of untreated **(S)** and NAC-treated granulomas from healthy individuals **(T)**. Data represent means ±SE from 8 healthy individuals. ^**^p < 0.005 when comparing samples at 8 and 15 days. ^*^p < 0.05 when comparing samples at 15 days with or without NAC treatment. ^**^p < 0.005 when comparing samples treated with NAC to the controls. ^*^p < 0.05 when comparing samples treated with NAC to the controls.

To build an *in vitro* model for human granulomas, we first infected PBMCs isolated from peripheral blood of healthy individuals with *M. tb* at MOI 0.1:1 and incubated for 15 days. PBMCs tended to form microscopic granulomas at 7-day time point and this was confirmed by staining the *in vitro* granulomas with Hematoxylin-Eosin. We observed more solidified and robust aggregation of cells in the granulomas treated with NAC compared to the untreated control group from healthy individuals (Figures 2C,D). NAC-treatment also resulted in a significant three-fold decrease in the intracellular survival of *M. tb* inside the granulomas compared to the untreated control group (Figure 2B).

To determine the underlying effector mechanisms against *M. tb* infection in NAC-treated granulomas, we quantified the levels of GSH, cytokines and the extent of phagosome-acidification. There was more than a two-fold increase in the levels of total GSH in NAC-treated granulomas compared to the untreated control group from healthy individuals (Figure 2E). Enhancement in the levels of GSH in NAC-treated granulomas was accompanied by a significant three-fold increase in the production of IFN-γ (Figure 2F). Phagosome acidification is considered to be one of the important effector mechanism that is responsible for intracellular killing of *M. tb*. NAC-treatment of granulomas also resulted in downregulation in the production of TNF-α. Measurement of TNF-α levels in the supernatants recovered from NAC-treated granulomas at 8 and 15 days indicated a two and three-fold decrease, respectively in the production of this cytokine (Figures 2G,H). In comparison to the untreated control group there was a statistically significant increase in the number of *M. tb* inside the acidified compartments in NAC-treated granulomas (Figures 2I,J). However, in untreated granulomas, bacteria were mostly observed inside the non-acidified compartments (Figure 2I). To examine the production of ROI in the granulomas cellROX staining was performed. Intense cellROX staining was observed in untreated granulomas (Figure 2K). NAC-treatment resulted in a decrease in the levels of ROI as evident from the diminished uptake of cellROX (Figure 2L). Propidium iodide staining was performed to determine the extent of necrosis in the granulomas. Increased necrosis was observed amongst the untreated granulomas (Figure 2M). NAC-treatment resulted in a decrease in the extent of necrosis (Figure 2N). Microscopic observation of the immunofluorescent staining indicate that the *in vitro* granulomas constitute cell types such as: macrophages, monocytes, dendritic cells CD4 and CD8 T cells all of which contribute to the innate and adaptive immune responses against *M*. *tb* infection (Figures 2O–T). An intense staining for CD4, CD8, and CD86 molecules was observed in NAC-treated granulomas suggesting that reduction in the extent of necrosis and ROI production will maintain the viability of myeloid and lymphoid cells in the granulomas (2L, N, P, R, T).

### *M. tb* survival and effector responses against the pathogen inside inh and INH+NAC-treated granulomas from healthy subjects

**Figure 3:**
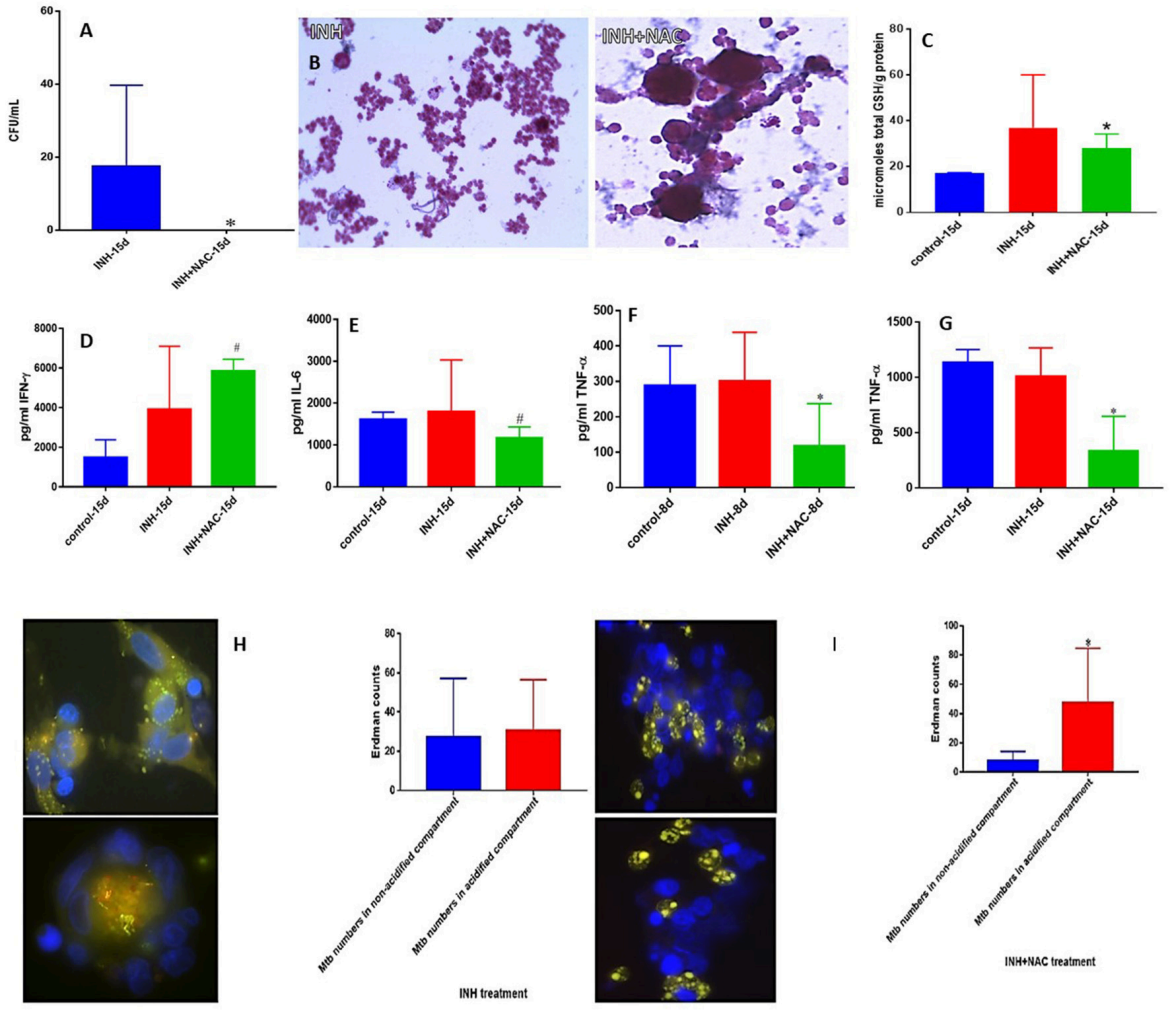
INH and INH+NAC effects on *in vitro* granulomas developed using immune cells from healthy subjects. Survival of *M. tb* inside INH and INH+NAC-treated granulomas **(A)**, Hematoxylin and Eosin staining of INH and INH+NAC-treated granulomas from healthy individuals **(B)**, determination of GSH levels at 15 days post-infection **(C)**, IFN-γ levels at 15 days post-infection **(D)**, IL-6 levels at 15 days post-infection **(E)**, TNF-α levels at 8 days **(F)** and 15 days **(G)** post-infection, and phagosome acidification at 15 days post-infection **(H,I)**. INH+NAC treatment resulted in a significant and complete clearance of *M. tb* infection inside the granulomas from healthy subjects compared to treatment with INH alone **(A)**. INH+NAC treatment also induced formation of solid and stable granulomas **(B)**. ^*^p < 0.05 when comparing samples treated with INH only to those treated with INH and NAC. ^#^*p* < 0.05 when comparing samples treated with INH and NAC to their controls.

INH is one of the most important first line antibiotic used in conjunction with other antibiotics to treat active TB. Treatment of *in vitro* granulomas with combination of NAC and INH resulted in total clearance of *M. tb* when compared to granulomas solely treated with INH alone (Figure 3A). INH+NAC treatment resulted in more numbers of solid and stable granulomas compared to granulomas treated only with INH (Figure 3B).

We then determined the effector responses against *M. tb* inside INH and INH+NAC treated granulomas from healthy individuals. There was a significant two-fold increase in the levels of total GSH in INH+NAC treated granulomas similar to the NAC treated category (Figure 3C). Increased levels of GSH in INH+NAC treated granulomas was accompanied by a significant three-fold increase in the production of IFN-γ (Figure 3D). Excessive production of pro-inflammatory cytokines, IL-6 and TNF-α is associated with oxidative stress and inflammation. INH+NAC treatment resulted in a significant decrease in the levels of IL-6 (Figure 3E). INH+NAC treatment also resulted in a significant decrease in the levels of TNF-α at both 8 days and 15 days (three-fold decrease) post-infection compared to untreated control category (Figures 3F,G). Importantly, INH+NAC treatment resulted in a five-fold increase in the number of *M. tb* inside the acidified compartments (Figure 3I). However, there was no notable increase in the number of bacteria in the acidified compartments in granulomas solely treated with INH (Figure 3H).

### *M. tb* survival and effector responses against the pathogen inside RIF and RIF+NAC-treated granulomas from healthy subjects

**Figure 4:**
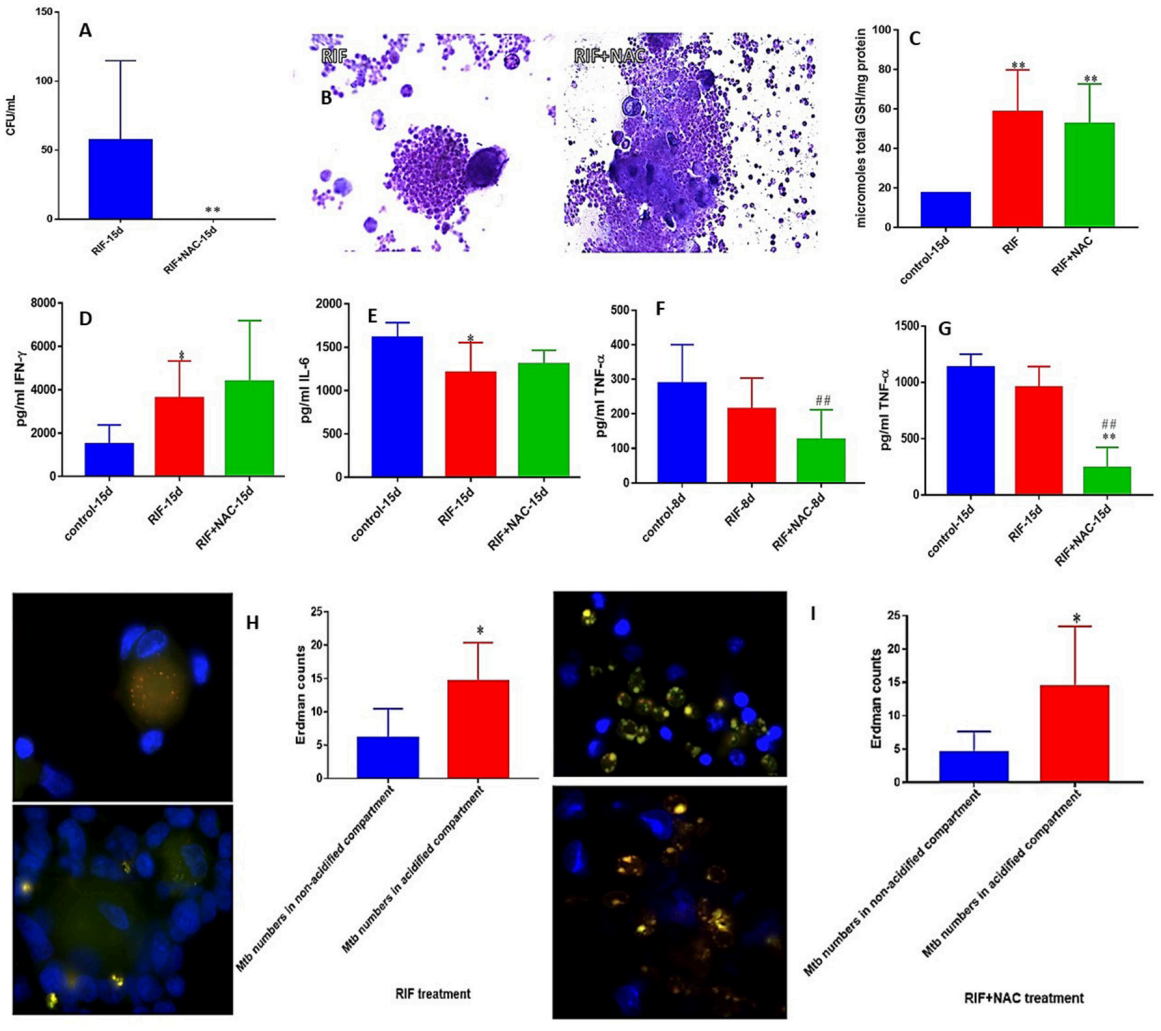
RIF and RIF+NAC effects on *in vitro* granulomas developed using immune cells from healthy subjects. Survival of *M. tb* inside RIF and RIF+NAC-treated granulomas **(A)**, Hematoxylin and Eosin staining of RIF and RIF+NAC-treated granulomas from healthy individuals **(B)**, determination of GSH levels at 15 days post-infection **(C)**, IFN-γ levels at 15 days post-infection **(D)**, IL-6 levels at 15d post-infection **(E)**, TNF-α levels at 8 days **(F)** and 15 days **(G)** post-infection, and phagosome acidification at 15 days post-infection **(H,I)**. ^*^p < 0.05 when comparing samples treated with RIF only to those treated with RIF and NAC. ^#^*p* < 0.05 when comparing samples treated with RIF and NAC to their controls. ^**^p < 0.005 when comparing samples treated with RIF only to those treated with RIF and NAC. ^*##*^*p* < 0.005 when comparing samples treated with RIF and NAC to their controls.

RIF is another important first line antibiotic generally used with INH to treat TB clinically. We observed a total clearance of *M. tb* in the granulomas treated with a combination of NAC and RIF when compared to sole treatment with RIF (Figure 4A). RIF+NAC treatment resulted in solid and dense aggregation of immune cells when compared to granulomas treated with RIF alone (Figure 4B).

We then determined the underlying mechanisms by which RIF+NAC-treatment augmented the effector responses against *M. tb* inside the granulomas from healthy individuals. We assayed the levels of total GSH in both RIF and RIF+NAC treated granuloma lysates. There was a significant three-fold increase in the levels of total form of GSH in both RIF and RIF+NAC treated granulomas compared to the untreated group (Figure 4C). Increased levels of GSH in both RIF and RIF+NAC treated granulomas were accompanied by a notable increase in the production of IFN-γ and decreased levels of IL-6 (Figures 4D,E). RIF+NAC treatment also resulted in a significant decrease in the levels of TNF-α at both 8 days and 15 days (three-fold decrease) post-infection compared to untreated control category (Figures 4F,G). Treatment of granulomas with RIF+NAC resulted in three-fold increase in the number of *M. tb* inside the acidified compartments (Figure 4I).

### *M. tb* survival and effector responses against the pathogen inside EMB and EMB+NAC-treated granulomas from healthy subjects

**Figure 5:**
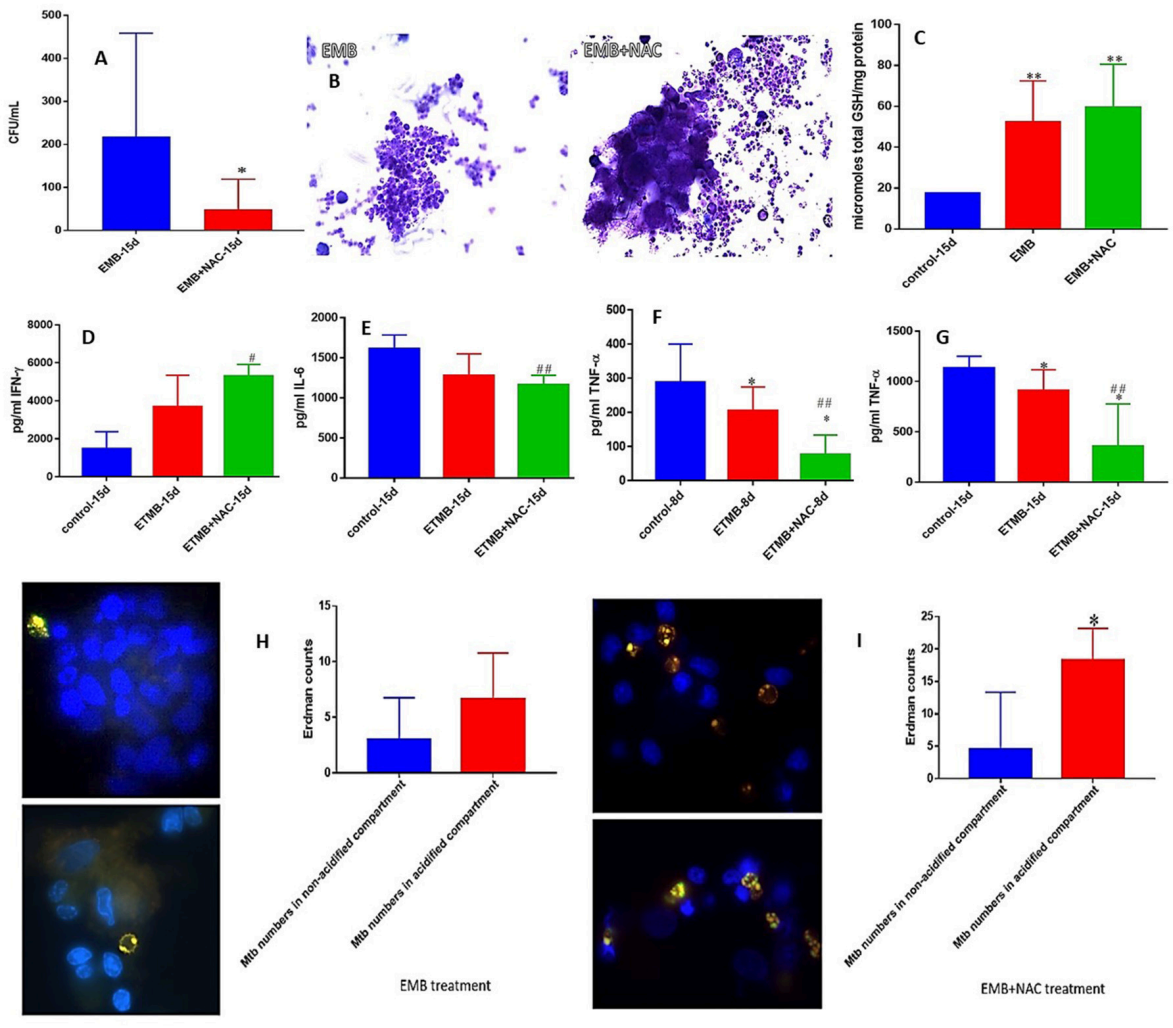
EMB and EMB+NAC effects on *in vitro* granulomas developed using immune cells from healthy subjects. Survival of *M. tb* inside EMB and EMB+NAC-treated granulomas **(A)**, Hematoxylin and Eosin staining of EMB and EMB+NAC-treated granulomas from healthy individuals **(B)**, determination of GSH levels at 15 days post-infection **(C)**, IFN-γ levels at 15 days post-infection **(D)**, IL-6 levels at 15 days post-infection **(E)**, TNF-α levels at 8 days **(F)** and 15 days **(G)** post-infection, and phagosome acidification at 15 days post-infection **(H,I)**. ^*^p < 0.05 when comparing samples treated with INH only to those treated with EMB and NAC. ^#^*p* < 0.05 when comparing samples treated with EMB and NAC to their controls. ^**^p < 0.005 when comparing samples treated with INH only to those treated with EMB and NAC. ^*##*^*p* < 0.005 when comparing samples treated with EMB and NAC to their controls.

EMB is also a first line antibiotic administered during the initial 2 months of TB treatment in combination with INH, RIF, and PZA. Although there was no complete clearance of bacterial infection, Treatment of granulomas with EMB+NAC resulted in a significant decrease in the number of *M. tb* when compared to granulomas solely treated with EMB (Figure 5A). EMB+NAC treatment also resulted in dense aggregates of immune cells forming stable granulomas (Figure 5B).

Consistent with our previous findings, EMB+NAC treatment resulted in a significant decrease in the levels of IL-6 (Figure 5E). We observed a significant decrease in the levels of TNF-α in EMB-treated granulomas at both 8 and 15 days post-infection (Figures 3G, 5F). Furthermore, EMB +NAC treatment resulted in further downregulation (three-fold decrease) and a significant decrease in the levels of TNF-α at both 8 and 15 days post-infection compared to untreated control category (Figures 3G, 5F). EMB+NAC treatment also resulted in a four-fold significant increase in the number of *M. tb* inside the acidified compartments (Figure 5I). We did not observe any increase in the number of *M. tb* inside the acidified compartments in granulomas solely treated with EMB (Figure 5H).

### Granulomas from individuals with t2dm, structure, the levels of cytokines and oxidative stress markers, and survival of *M. tb* inside untreated and NAC-treated granulomas

**Figure 6:**
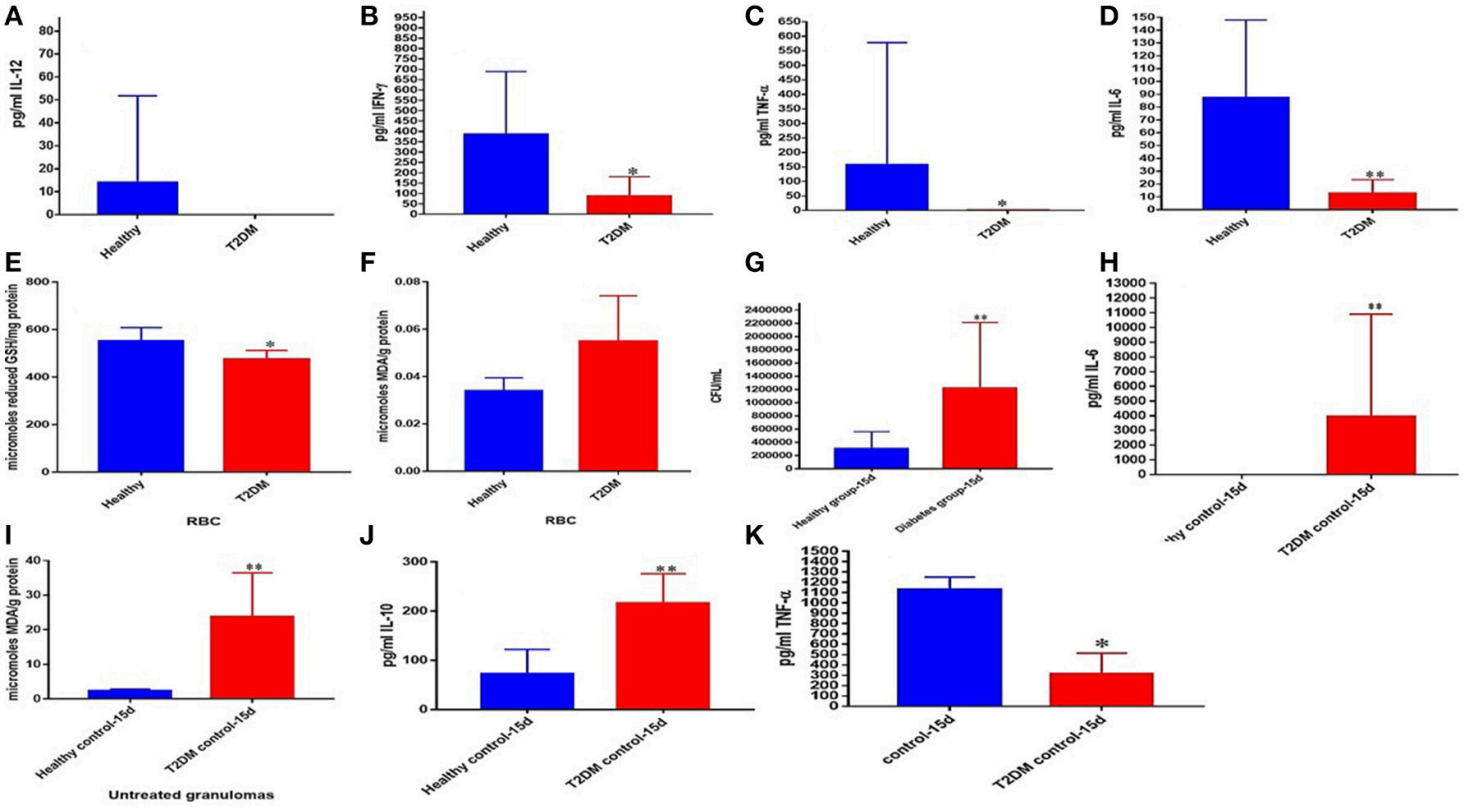
Comparison of systemic markers and granulomas from healthy individuals and T2DM individuals. Levels of IL-12 **(A)**, IFN-γ **(B)**, TNF-α **(C)**, and IL-6 **(D)** in the plasma samples of healthy and T2DM individuals. Levels of GSH **(E)** and MDA **(F)** in red blood cells of healthy and T2DM individuals. Comparison of *M. tb* survival in healthy individuals and participants with T2DM **(G)**. Levels of IL-6 **(H)**, MDA **(I)**, IL-10 **(J)** and TNF-α **(K)** in granuloma supernatants from healthy subjects and individuals with T2DM. ^*^p < 0.05 when comparing samples of healthy individuals to those with T2DM. ^**^p < 0.005 when comparing samples of healthy individuals to those with T2DM.

**Figure 7:**
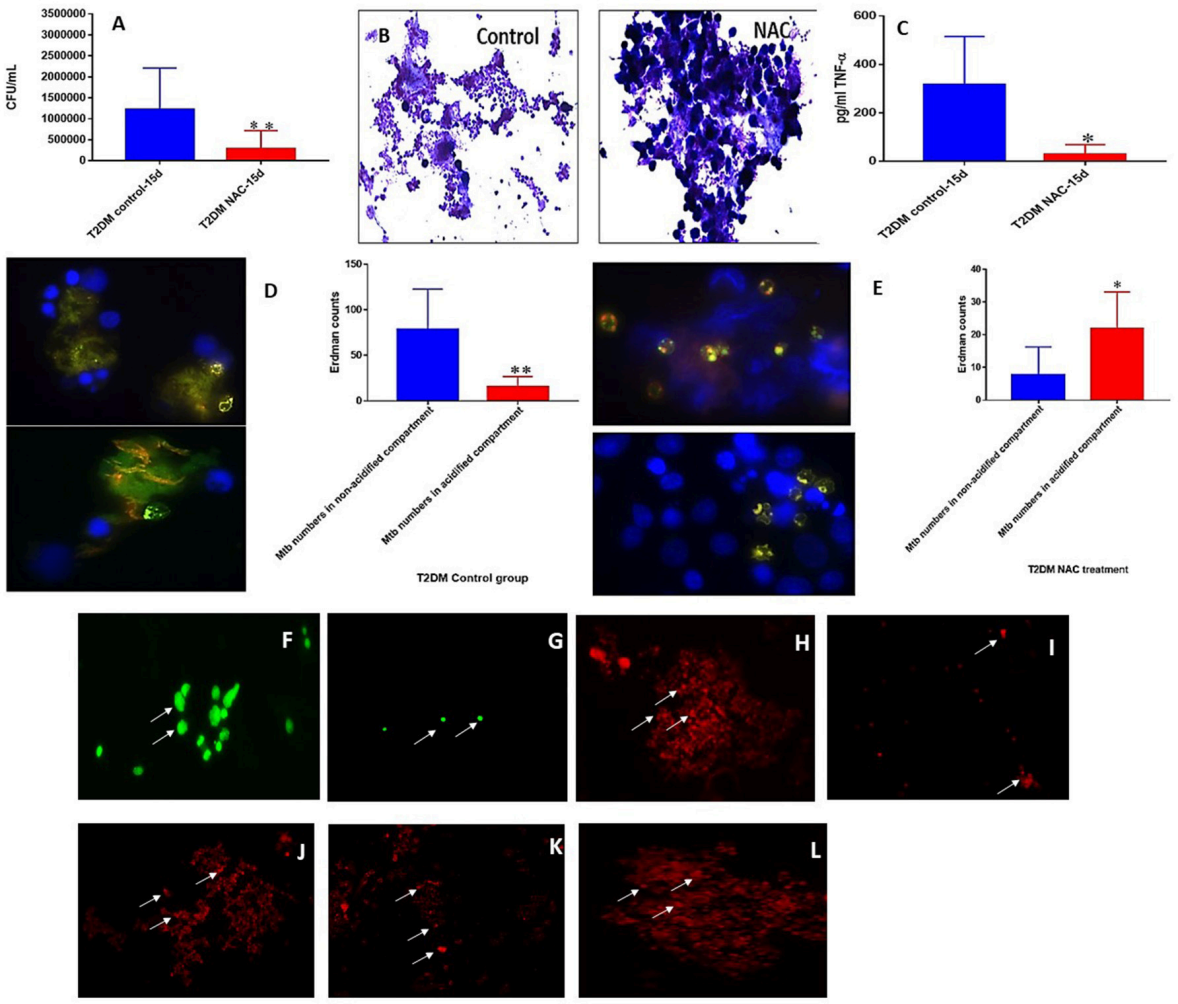
NAC effects on *in vitro* granulomas developed using immune cells from T2DM subjects. *M. tb* survival in untreated and NAC treated granulomas from individuals with T2DM **(A)**. Hematoxylin and eosin staining of untreated and NAC treated granulomas from T2DM individuals **(B)**. Assay of in TNF-α in supernatants form untreated and NAC treated granulomas from individuals with T2DM **(C)**. Quantification of phagosome acidification in untreated **(D)** and NAC **(E)** treated granulomas from individuals with T2DM. cellROX staining of untreated **(F)** and NAC-treated granulomas **(G)** from individuals with T2DM. Propidium iodide staining of untreated **(H)** and NAC-treated granulomas **(I)** from individuals with T2DM. CD4 staining of untreated granulomas **(J)** from individuals with T2DM. CD8 staining of untreated granulomas **(K)** from individuals with T2DM. CD86 staining of untreated granulomas **(L)** from individuals with T2DM. ^*^p < 0.05 when comparing healthy to T2DM. ^**^p < 0.005 when comparing healthy to T2DM.Microscopy work was done with a light microscope at 1000x magnification under oil immersion.

We also attempted to characterize the effector immune responses inside the *in vitro* granulomas from individuals with T2DM. We observed a significant decrease in the levels of TNF-α, IFN-γ, IL-12, and IL-6 in the baseline plasma samples from individuals with T2DM indicating a systemic downregulation in the cytokine levels among uncontrolled diabetes (Figures 6A–D). Decreased levels of GSH along with increased levels of malondialdehyde (MDA) in the red blood cells (RBCs) serve as an important marker for systemic oxidative stress. GSH levels in the RBCs isolated from individuals with T2DM were significantly diminished compared to the healthy subjects (Figure 6E). Decreased levels of GSH in the RBCs isolated from individuals with T2DM correlated with increased levels of MDA (Figure 6F). *In vitro* granulomas generated from PBMCs isolated from individuals with T2DM were not as robust and solid as the healthy individuals and this observation correlated with impaired ability of granulomas to control *M. tb* infection. Granulomas from individuals with T2DM were found to harbor significantly more intracellular *M. tb* than their healthy counterparts (Figure 6G). Increased survival of *M. tb* also correlated with a significant increase in the levels of MDA and IL-6 in granulomas from individuals with T2DM individuals (Figures 6G–I) as well as a decrease in TNF-α levels (Figure 6K). NAC-treatment resulted in a significant four-fold decrease in the intracellular survival of *M. tb* inside the granulomas from individuals with T2DM (Figure 7A). Although NAC-treatment improved cell aggregation, nonetheless, granulomas from individuals with T2DM were not as solid as those from healthy subjects (Figure 7B). NAC-treatment of granulomas from individuals with T2DM also resulted in a significant decrease in the levels of TNF-α (Figure 7C). There was a significant increase in the number of *M. tb* in the non-acidified compartments in untreated granulomas from individuals with T2DM (Figure 7D). In contrast to the untreated-treated granulomas there was a significant increase in the number of *M. tb* inside the acidified compartments of NAC-treated granulomas from individuals with T2DM (Figure 7E). An intense cellROX staining was observed in untreated granulomas from T2DM group, indicative of oxidative stress (Figure 7F). Treatment of granulomas from T2DM group with NAC resulted in a decrease in the levels of ROI as evident from the diminished uptake of cellROX (Figure 7G). Increased uptake of Propidium iodide was also observed in the untreated granulomas from T2DM group (Figure 7H). NAC-treatment of granulomas from T2DM group resulted in a decrease in the extent of necrosis (Figure 7I). The cell types that constituted *in vitro* granulomas from T2DM group were macrophages, monocytes, dendritic cells CD4 and CD8 T cells (Figures 7J–L).

### *M. tb* survival and effector responses against the pathogen inside INH and INH+NAC-treated granulomas from subjects with T2DM

**Figure 8:**
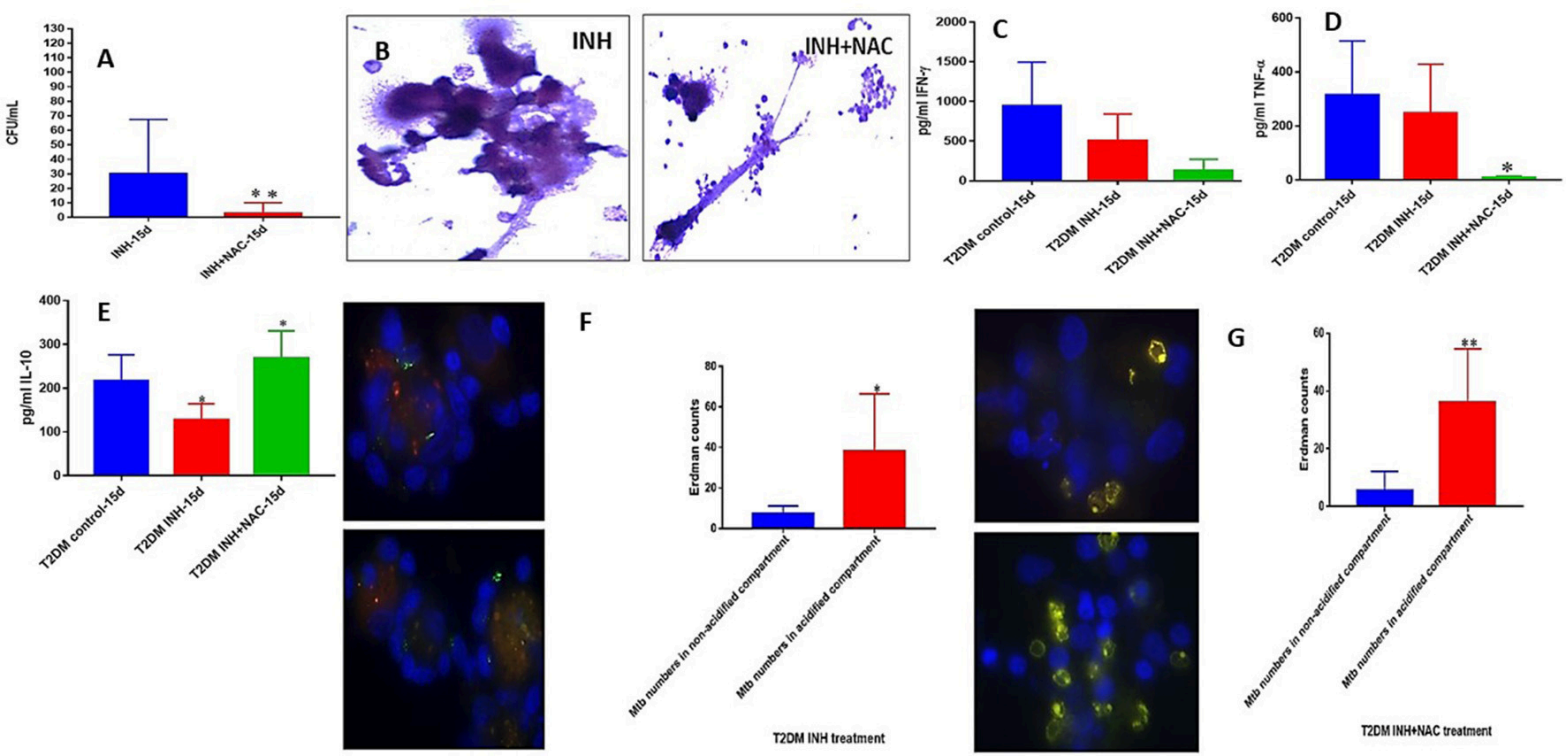
INH and INH+NAC effects on *in vitro* granulomas developed using immune cells from individuals with T2DM. Survival of *M. tb* inside INH and INH+NAC-treated granulomas **(A)**, Hematoxylin and Eosin staining of INH and INH+NAC-treated granulomas from T2DM individuals **(B)**, IFN-g levels at 15 days post-infection **(C)**, TNF-a levels at 15 days post-infection **(D)**, IL-10 levels at 15 days **(E)** post-infection, and phagosome acidification at 15 days post-infection **(F,G)**. ^*^p < 0.05 when comparing samples treated with INH+NAC to controls. ^**^p < 0.005 when comparing samples treated with INH+NAC to controls.

There was a significant ten-fold reduction in the survival of *M. tb* inside INH+NAC treated granulomas from subjects with T2DM compared to granulomas treated with INH alone (Figure 8A). However, in contrast to healthy subjects, INH+NAC treatment of granulomas from T2DM group did not result in complete clearance of *M. tb* (Figures 3A, 8A). In contrast to the healthy group, INH+NAC treated granulomas from the T2DM group did not form solid and stable granulomas (Figure 8B).

In order to test whether the trend in the effector responses against *M. tb* in INH+NAC granulomas from T2DM group is similar to the healthy counterpart, we measured the levels of cytokines and phagosome acidification. A distinct and significant decrease in the levels of TNF-α was observed in the INH+NAC treated granulomas compared to both untreated and INH treated counterparts in granulomas from subjects with T2DM (Figure 8D). NAC-treatment of granulomas from T2DM group did not result in the restoration in the levels of GSH (data not shown). In contrast to healthy subjects, treatment of granulomas from subjects with T2DM with INH+NAC did not result in an increase in the levels of IFN-γ. On the contrary, there was an observable decrease in the levels of IFN-γ in the INH and INH+NAC treated granulomas from subjects with T2DM compared to that in the untreated counterpart (Figure 8C). INH+NAC treatment of granulomas from subjects with T2DM also resulted in a significant increase in the production of IL-10 (Figure 8E). Treatment of granulomas from individuals with T2DM with INH and INH+NAC resulted in significant number of *M. tb* numbers inside the acidified compartments (Figures 8F,G). Our results signify that NAC-treatment has direct antimycobacterial effects, can reduce the levels of TNF-α, and can synergistically enhance the effects of INH in reducing the bacterial burden. However, INH+NAC treatment of granulomas from subjects with T2DM resulted in decrease in the levels of IFN-γ and increase in the production of IL-10.

### *M. tb* survival and effector responses against the pathogen inside RIF and RIF+NAC-treated granulomas from subjects with T2DM

**Figure 9:**
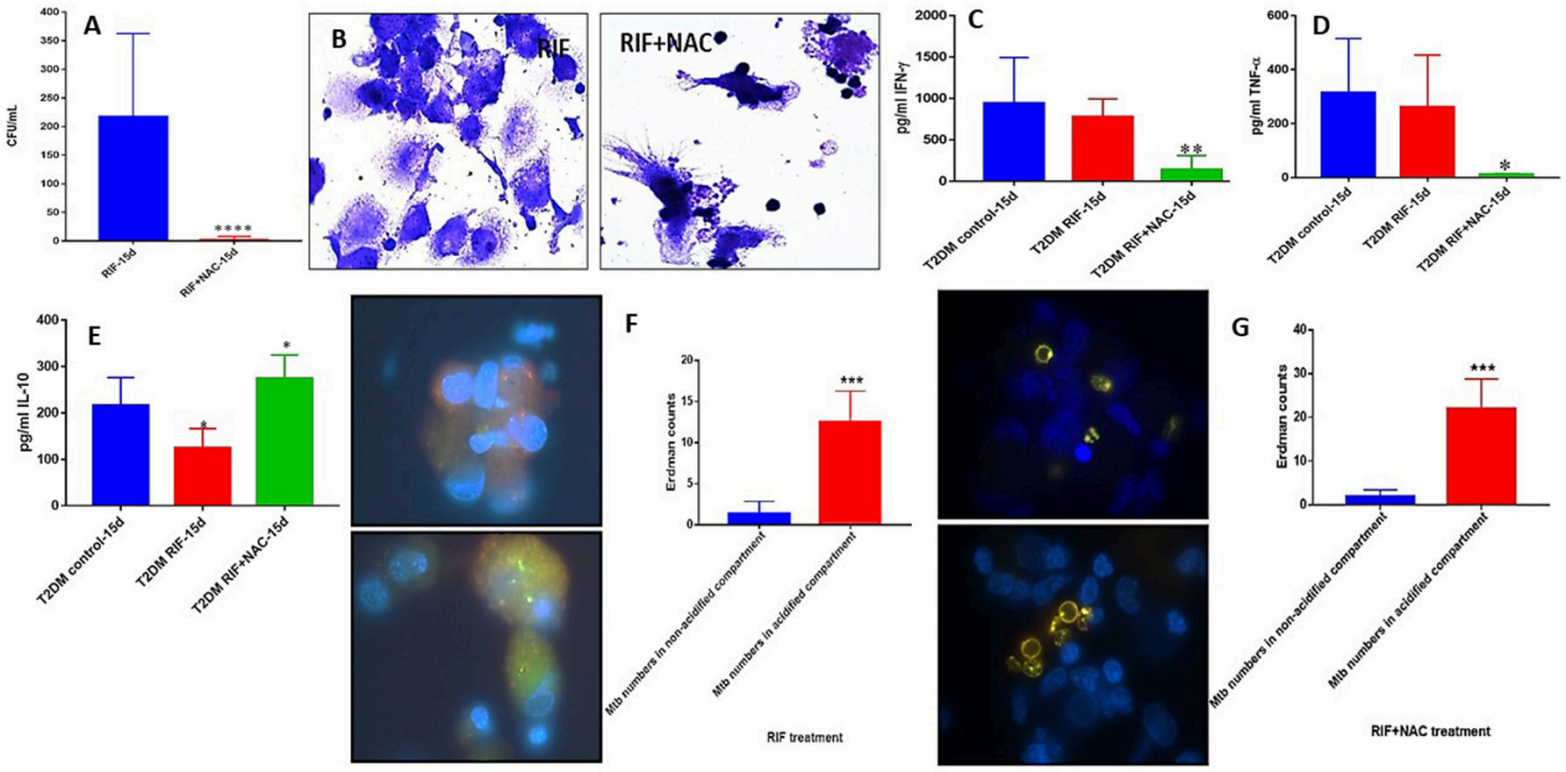
RIF and RIF+NAC effects on *in vitro* granulomas developed using immune cells from individuals with T2DM. Survival of *M. tb* inside RIF and RIF+NAC-treated granulomas **(A)**, Hematoxylin and Eosin staining of RIF and RIF+NAC-treated granulomas from T2DM individuals **(B)**, IFN-g levels at 15d post-infection **(C)**, TNF-a levels at 15 days post-infection **(D)**, IL-10 levels at 15 days **(E)** post-infection, and phagosome acidification at 15 days post-infection **(F,G)**. ^****^p < 0.00005 when comparing samples treated with RIF only to those treated with RIF and NAC. ^*^p < 0.05 when comparing samples treated with RIF+NAC to controls. ^**^p < 0.005 when comparing samples treated with RIF+NAC to controls. ^***^p < 0.0005 when comparing samples treated with RIF+NAC to controls.

There was a significant hundred-fold decrease in the number of *M. tb* inside the RIF+NAC treated granulomas from subjects with T2DM, compared to treatment with RIF alone (Figure 9A). Consistent with INH+NAC treatment, treatment of granulomas from T2DM group with RIF+NAC did not result in the formation of robust granulomas (Figure 9B).

Treatment of granulomas from T2DM subjects with RIF+NAC resulted in a significant decrease in the levels of IFN-γ (Figure 9C) and TNF-α (Figure 9D), and a significant increase in the levels of IL-10 (Figure 9E). These results are similar to the findings from INH+NAC treatment in the diabetic group. Treatment of T2DM granulomas with RIF alone resulted in a significant and six-fold increase in the number of *M. tb* in the acidified compartments (Figure 9F). Furthermore, treatment with RIF+NAC resulted in a significant and ten-fold increase in the number of *M. tb* in the acidified compartments (Figure 9G) indicating that NAC has direct effects in promoting phagosome-lysosome fusion. Inability of NAC to enhance the levels of IFN-γ and decrease the levels of IL-10 in granulomas from individuals with T2DM could directly be linked to GSH insufficiency since NAC cannot effectively restore the levels of GSH due to diminished levels of GSH *de novo* synthesis enzymes in the diabetic group.

### *M. tb* survival and effector responses against the pathogen inside EMB and EMB+NAC-treated granulomas from subjects with T2DM

**Figure 10:**
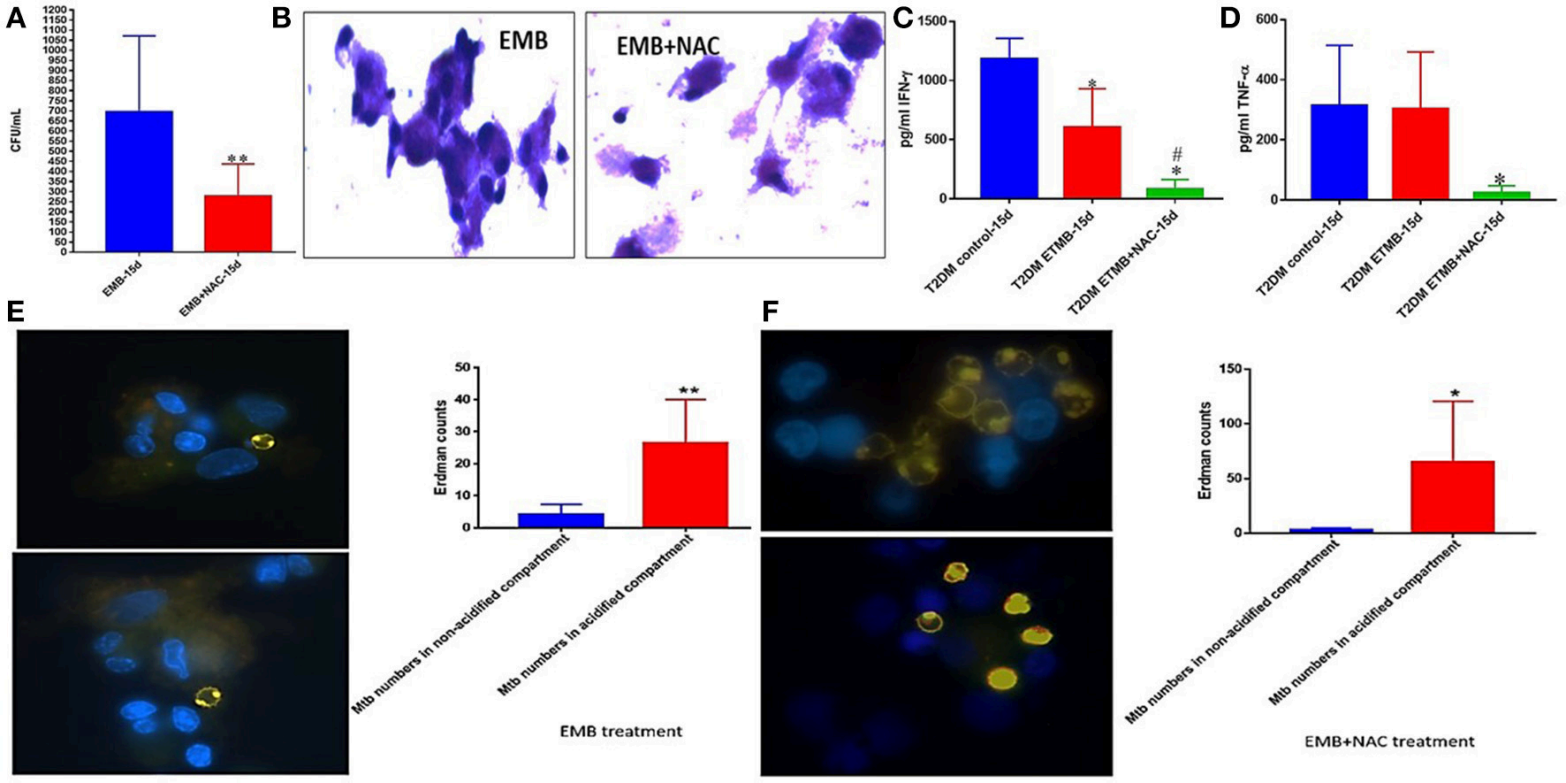
EMB and EMB+NAC effects on *in vitro* granulomas developed using immune cells from individuals with T2DM. Survival of *M. tb* inside EMB and EMB+NAC-treated granulomas **(A)** Hematoxylin and Eosin staining of RIF and RIF+NAC-treated granulomas from T2DM individuals **(B)**, IFN-g levels at 15d post-infection **(C)**, TNF-a levels at 15 days post-infection **(D)**, and phagosome acidification at 15 days post-infection **(E,F)**. ^**^p < 0.005 when comparing samples treated with EMB only to those treated with EMB and NAC. ^*^p < 0.05 when comparing samples treated with EMB+NAC to controls. ^**^p < 0.005 when comparing samples treated with EMB+NAC to controls. ^#^*p* < 0.05 when comparing samples treated with EMB+NAC to controls.

EMB+NAC treatment resulted in a significant and approximately three-fold reduction in the viability of *M. tb* inside the granulomas from subjects with T2DM (Figure 10A). EMB+NAC-treatment did not enhance the formation of solid and stable aggregation of immune cells (Figure 10B).

Treatment of granulomas from individuals with T2DM with EMB or EMB+NAC resulted in a significant reduction in the levels of IFN-γ (Figure 10C). EMB+NAC treatment of granulomas from individuals with T2DM also resulted in a significant reduction in the levels of TNF-α (Figure 10D). Treatment of T2DM granulomas with EMB alone resulted in a significant and six-fold increase in the number of *M. tb* in the acidified compartments (Figure 10E). Importantly, treatment with EMB+NAC resulted in a significant and approximately twelve-fold increase in the number of *M. tb* in the acidified compartments (Figure 10F).

## Discussion

Worldwide, TB is considered to be the leading cause of mortality due to a single infectious agent (1). Furthermore, immunocompromised individuals, such as those with uncontrolled diabetes, are increasingly susceptible to *M. tb* infection (9). There exists abundant evidence which affirms that patients with diabetes are at a higher risk for developing an active TB infection, which is commonly believed to be by means of a diminished cell-mediated immunity (10, 11, 48–50). The conventional method for *M. tb* abolition is with the standard 6-month antibiotic course of treatment (51).

Our lab has formerly reported that H37Rv, the virulent laboratory-strain of *M. tb*, is vulnerable to GSH when supplemented i*n vitro* (28, 52). Likewise, we have established that enhancing the levels of GSH through treatment with NAC inhibits the intracellular survival of H37Rv (28, 29, 36, 53). Therefore, GSH possess direct antimycobacterial potentiality, which assists human macrophages in the innate defense against *M. tb* infection. Furthermore, our lab has previously revealed that the levels of intracellular GSH are significantly abridged among individuals with T2DM, in consequence to compromised levels of the enzymes involved in GSH synthesis (33, 34, 54–57).

Accordingly, we investigated the synergistic efficacy of the first-line antibiotics INH, RIF, and EMB administered at sub-optimal levels in conjunction with NAC, the GSH precursor, on PBMC-derived *in vitro* granulomas; processed from the blood of both healthy and T2DM individuals and infected with the Erdman strain of *M. tb*. This study focuses on the mechanistic actions of immune cells during an *M. tb* infection within granulomas, to illuminate their subsequent immune activity in the uniquely regulated environment. Our *in vitro* granulomas constitute cell types such as: macrophages, monocytes, dendritic cells, CD4 and CD8T cells all of which contribute to the immune responses necessary for granuloma formation in consequence to *M. tb* infection.

We first examined the rate of growth or mortality of *M. tb* in 7H9 media, in the absence immune cells, when sham treated, supplemented with the various standalone antibiotics or in combination with lone antibiotics and the GSH antecedent NAC. We observed a roughly 1-log growth of the mycobacteria when it was left untreated (Figure 1A). This confirms the ability of Erdman strain of *M. tb* to actively replicate in the absence of therapeutics. Significantly, when the immunoadjuvant NAC was administered, over a 5-log reduction in the viability of *M. tb* was observed (Figure 1B). These results support previous findings which indicate that NAC possess mycobactericidal capacity (35, 39, 43). Furthermore, all three first line antibiotics investigated showed a drastic bacterial reduction of statistical significance compared to the controls, and when NAC was co-administered with the lone antibiotics a further statistically significant diminution was detected (Figures 1C–E).

We next reproduced the aforestated experimental applications on *in vitro* derived granulomas emanated from the PBMCs of healthy individuals. Similar to the previous experiment without immune cells present, we observed a statistically significant increase in *M. tb* abundance, when the cells were not accompanied by treatment additives (Figure 2A). Enhancing the levels of GSH in healthy PBMCs, by NAC application, induced significant inhibition in the intracellular growth of *M. tb* (Figure 2B). This data further demonstrates NAC's (GSH's) antimycobacterial activity, and functionality in the immune defense against *M. tb*. A vehement decrease in mycobacterial survival was again observed after each respective antibiotic was administered to the cells (Figures 3A, 4A, 5A). As expected, each antibiotic delivered at its MIC caused a drastic reduction of viable *M. tb*, and outright mycobacterial elimination was not observed. However, when each individual antibiotic was administered in combination with NAC, a statistically significant decrease in mycobacterial quantity was observed in comparison to the treatment of uNACcompanied antibiotics (Figures 3A, 4A, 5A). Importantly, complete *M. tb* clearance was detected following the combined treatment of both INH and NAC, as well as RIF and NAC (Figures 3A, 4A). This evidence suggests that the combined treatment of various first-line antibiotics supplemented with NAC (GSH) encompasses greater prophylactic efficacy than either of the treatments given unassisted. Therefore, the synergistic effects of NAC (GSH) and antibiotics grant improved innate immune control over intracellular *M. tb* and advocates GSHs therapeutic capability as an adjunct with first-line antibiotics in clearing a *M. tb* infection.

We have previously reported that *M. tb* infection can cause considerable depletion of intracellular GSH compared to uninfected cells, which in turn can promote *M. tb* survival (34, 54–57). When NAC was administered to the granulomas of healthy individuals, a statistically significant increase of GSH was witnessed (Figure 2E). These results affirm that the addition of NAC, GSH's forerunner, will indeed cause the levels of GSH to become subsequently elevated in response. Enhancing the levels of GSH with NAC treatment, in *M. tb* infected cells appropriately displays an inverse correlation to the significant reduction of *M. tb* intracellular survivability (Figures 2A,E). The treatment of *M. tb* granulomas with each of the first-line antibiotics resulted in a significant upsurge of GSH levels (Figures 3C, 4C, 5C). We believe that the increase in GSH observed after the treatment with lone antibiotics is due to a restorative effect, which is most likely as a result of the antibiotics diminishing the *M. tb* bio-burden thus enabling the host cells to restore the levels of GSH which further improves their ability to combat the infection. Likewise, treatment of the granulomas with each of the antibiotics in concurrence with NAC resulted in the elevation of GSH for all three categories, all of which presented a statistically significant increase in relation to the control group (Figures 3C, 4C, 5C).

An effective innate immune response against *M. tb* infection is established by virtue of the efficient regulation of immune cells, mediated by the release of cytokines (58, 59). The cytokine IFN-γ is crucial in both innate and adaptive immunity, serving as a catalyst for macrophage activation, and MHC molecule expression (60). Additionally, IFN-γ possesses immunostimulatory properties which induce effector mechanisms pivotal not only toward the activation of macrophages, but aid in granuloma formation, and promote macrophage phagosome-lysosome fusion, thus provoking enhanced *M. tb* infection management (61, 62). The successive supplementation of NAC to the granulomas from healthy subjects resulted in the statistically significant increase in the levels IFN-γ (Figure 2F). Likewise, each standalone antibiotic induced an IFN-γ release (Figures 3D, 4D, 5D). Importantly, a marked increase in IFN-γ was also detected for all of the antibiotic categories co-supplemented with NACcompared to their standalone counterparts (Figures 3D, 4D, 5D). This data reveals that again a restorative effect is displayed once the antibiotics have been administered to *M. tb* granulomas as distinguished by the elevation in IFN-γ released from the cells. Similarly, the profound amplification of IFN-γ after lone NAC supplementation and the co-supplementation of antibiotics administered with NAC, reveals the mechanistic potentiality for which these additives exert their prophylactic measures.

It has previously been shown that a decrease in the levels of proinflammatory cytokines such as IL-6, and an increase in immunosuppressive cytokines such as IL-10 can be immensely beneficial in limiting the pathology of a *M. tb* infection once the appropriate granulomatous response has taken place (30). IL-6 is a proinflammatory cytokine generated in response to factors which create cellular damage such as infections and injured tissues (63). However, persistent IL-6 production or overexpression can lead to the development of various diseases particularly those related to chronic inflammation and autoimmunity (64). Furthermore, it has been shown that IL-6 can inhibit the cytokine IFN-γ during a *M. tb* infection (65). Therefore, it has been suggested that virulent mycobacteria purposefully upregulate IL-6 production to combat innate immunity (65). A reduction in IL-6 was detected among all the antibiotic categories when supplemented in conjunction with NAC; furthermore, statistical significance was recognized for the addition of NAC to INH and EMB. This reduction in IL-6 illustrates how NAC administration with first-line antibiotics can impede *M. tb*'s targets of immune suppression, as well as aid in the downregulation of pathological nonspecific damage to host cells due to overactive proinflammatory defenses during the *M. tb* infection.

Antibiotic treatment given in conjunction with NAC also resulted in decreased production of TNF-α. TNF-α, another proinflammatory cytokine, is primarily produced by macrophages, and is essential in fostering the formation and maintenance of a granuloma in both acute and chronic phases of infection (66–69). However, the overexpression of TNF-α also been connected to many inflammatory and autoimmune diseases such as rheumatoid arthritis, inflammatory bowel disease, ankylosing spondylitis, and psoriasis (70, 71). Additionally, elevated levels of TNF-α have been shown to incite pulmonary tissue damage during host defense against *M. tb* infection (71–73). The TNF-α levels for each first-line antibiotic category given in combination with NAC was significantly reduced (Figures 3F,G, 4F,G, 5F,G). Likewise, when NAC was administered alone, a significant reduction in the levels of TNF-α was observed (Figures 2G,H). Interestingly, the levels of TNF-α detected from the lone NAC category directly corresponds with the relative levels of TNF-α observed among the antibiotic categories given in combination with NAC (Figures 2G,H, 3F,G, 4F,G, 5F,G). This suggests the reduction in TNF-α observed is primarily due to NAC's incorporation. Therefore, our results reveal that NAC treatment modulates the levels of TNF-α, which we believe to be sufficient enough to arrest hyperactive cellular inflation thus limiting tissue damage, yet potent enough to maintain a healthy granuloma. Altogether, the aforestated cytokine data advocates that NAC/GSH supplementation can assist the innate and adaptive immune responses against *M. tb* by means of cytokine modulation, which ultimately translates to increased mycobactericidal ability and enhanced control over the pathogen (Supplemental Figure 1).

We then performed microscopic analysis of the stained granulomas to visualize how the modified effector mechanisms alter the granulomatous structure in response to the various treatment categories. When NAC was administered to the infected cells, we observed more solid and stable granulomas compared to the control categories (Figures 2C,D). Similarly, in relation to lone antibiotic treatment, the antibiotic categories which were supplemented with NAC displayed not only a marked increase in cellular size, but the cellular density appears increased as well (Figures 3B, 4B, 5B). These observations indicate that NAC supplementation promotes formation of more substantial and concentrated granulomas. This advancement in granulomatous composition inversely correlates with extent of bacterial survival discussed previously. This signifies that the cellular enhancement observed after NAC treatment advocates comprehensive control over *M. tb* infection.

An important effector mechanism involved in the control of *M. tb* infection is phagolysosomal fusion within macrophages, wherein phagosomes fuse with an acidic lysosome causing the breakdown of the contents inside the compartment. We therefore quantified this mycobactericidal mechanism with fluorescent imaging by staining for acidified cellular compartments and examining where the GFP labeled bacteria reside. In the absence of NAC, *M. tb* tend to be loosely sequestered around the nuclei of the granulomatous cell aggregations, and acidification is relatively low due to the inexistent of red and the overlapping yellow color detected (Figures 2I, 3H, 4H, 5H). Conversely, the addition of lone NAC, or NAC to the antibiotic categories causes a drastic alteration in intracellular morphology and bacterial locality (Figures 2I, 3I, 4I, 5I). The inclusion of NAC causes the bacteria to become exceedingly more compartmentalized within the granulomas, and the magnitude of phagolysosomal acidification is augmented as well, as illustrated by the portion of red, and yellow colored bacteria (Figures 2, 3I, 4I, 5I). These assays further demonstrate that the addition of NAC promotes intracellular killing of mycobacteria, by depicting an increased quantity of phagolysosomal fusion within the *M. tb* occupied macrophages.

Before we performed the same experimental method on individuals with T2DM, we first examined the RBCs and plasma samples of the healthy and T2DM participants for baseline comparison. We observed that prior to *M. tb* infection, the plasma cytokines IL-12, IFN-γ, TNF-α, and IL-6 were all drastically reduced among the T2DM group compared to the healthy individuals (Figures 6A–D). These results are consistent with previous findings which ascertain that individuals with T2DM present dysregulation of normal cytokine production (54, 74). Therefore, this dysregulation may impede host defense mechanisms and result in the altered immune responses observed during *M. tb* infection. Next, we tested the RBCs, and found that the individuals with T2DM displayed significantly compromised levels of GSH as well as elevated levels of malondialdehyde (MDA) (Figures 6E,F). MDA is a byproduct of lipid peroxidation and is therefore used as a descriptive measurement of oxidative stress. This inverse relationship between the levels of GSH and MDA is to be expected, which reveals that as the levels of GSH dwindle among the diabetic individuals, their redox homeostasis becomes subverted as well, resulting in increased systemic oxidative stress.

Compared to the healthy controls there was a statistically significant increase in the total *M. tb* numbers and the levels of IL-6, IL-10 and MDA, in granulomas from the T2DM group (Figures 6G–J). This data demonstrates superior bacterial viability emanating from diabetic cells, which is indicative of a worse prognosis; and further advocated by increased inflammation due to elevated IL-6, and greater oxidative damage represented by inflated MDA levels. However, the supplementation of NAC to T2DM-granulomas resulted in a statistically significant reduction of nearly two-thirds the bacterial load (Figure 7A). This advancement as a result of NAC supplementation is accompanied by a moderate increase in granulomatous size and density, as well as augmented phagolysosomal fusion as observed from the microscopic imaging, when compared to the sham-treated controls (Figures 7B,D,E). This data suggests that similar to the healthy individuals, treatment of T2DM samples with NAC results in enhanced prophylactic *M. tb* control at the cellular level. When the samples from T2DM subjects were treated with antibiotics, a significant reduction was revealed similar to that of the healthy individuals, although the bacterial quantity, and thus viability was elevated in comparison to the treatment of healthy subjects (Figures 8A, 9A, 10A). Likewise, the co-administration of NAC and antibiotics resulted in a statistically significant reduction compared to the lone antibiotics, though the bacterial abundance was greater than the healthy subjects, and complete clearance was no longer observed for the INH or RIF categories co-treated with NAC (Figures 8A, 9A, 10A). The fluorescent imaging of the antibiotic medicant categories illustrates that the incorporation of NAC into treatment significantly enhances the phagolysosomal fusion (Figures 8F,G, 9F,G, 10E,F). This data affirms that NAC supplementation improves mycobactericidal proficiency, and aids in infection ascendancy for both healthy and T2DM individuals. However, the increased cytokine production of IFN-γ observed among the healthy individuals subsequent to the administration NAC was not detected amongst the T2DM subjects (Figures 8C, 9C, 10C). Additionally, the levels of total GSH did not increase due to NAC supplementation among the T2DM subjects either (data not shown). Similarly, a preceding study reported that after oral NAC supplementation, the levels of *in vivo* GSH did not improve among T2DM patients (75). We suspect that this is due to the fact that T2DM individuals have attenuated levels of GSH synthesizing enzymes, and therefore despite elevating NAC (the GSH precursor), they are not capable of upregulating the extent of GSH produced (54). Additionally, the H&E imaging of the T2DM samples reveal that unlike the former experimental findings, the addition of NAC to the antibiotic treatment categories does not result in more solid stable granuloma formation (Figures 8B, 9B, 10B). We suspect that the reason the antibiotics co-administered with NAC do not form stable granulomas is due to the insufficient quantity of TNF-α present after NAC supplementation (Figures 8D, 9D, 10D). Unlike the aforementioned experimental conditions, the levels of TNF-α are already depressed amongst the T2DM subjects, comparable in measure to the healthy individuals after NAC supplementation. Therefore, the additional evanescence of TNF-α in consequence to NAC administration results in an inadequate supply for maintaining appropriate granuloma formation.

These novel findings illustrate that adding NAC to antibiotic treatment is capable of eliciting significant improvement in the granulomatous and mycobactericidal responses against a *M. tb* infection (Supplemental Figures 1, 2). Our results demonstrate that the addition of NAC results in significant reduction of *M. tb* burden in both healthy and diabetic individuals. Furthermore, in healthy individuals, NAC promotes the formation more solid and stable granulomas, as well as increased acidification of *M. tb* inhabited phagosomes. Accordingly, our results indicate that NAC can be advantageous as a prophylactic adjunct to first-line antibiotics, bolstering cytokine modulation, as well as the reduction and clearance of *M. tb* infection. Therefore, we believe that enhancing GSH by means of NAC supplementation in the antibiotic treatment of TB would not only reduce the toxicity of anti-TB medications through GSH's redox potentiality, but permit lessening the required antibiotic dosages to confer mycobacterial clearance, which could promote enhanced treatment compliance and circumvent the emergence of additional strains of DR-TB.

## Author contributions

The studies were conceived by the corresponding author, VV. VV designed the studies and mentored his lab members to conduct the studies and draft the manuscript. VV analyzed the data. GT and RC equally contributed to the work by performing all the experiments. GT was actively involved in drafting the manuscript. HI conducted the experiments along with GT and RC, AM, and AS recruited healthy subjects and participants with type 2 diabetes for this study. RP and CP provided technical support for microscopy. MF and LZ provided partial funding support and guidance to the students, respectively. SS provided Erdman strain of M.tb and expertise advise on granuloma imaging.

### Conflict of interest statement

The authors declare that the research was conducted in the absence of any commercial or financial relationships that could be construed as a potential conflict of interest.
