# Supplementary figures and images for: The Synergistic Effects of the Glutathione Precursor, NAC and First-Line Antibiotics in the Granulomatous Response Against Mycobacterium tuberculosis
Source: Front Immunol. 2018 Sep 12;9:2069. doi: 10.3389/fimmu.2018.02069 (PMC6144952; doi:10.3389/fimmu.2018.02069)

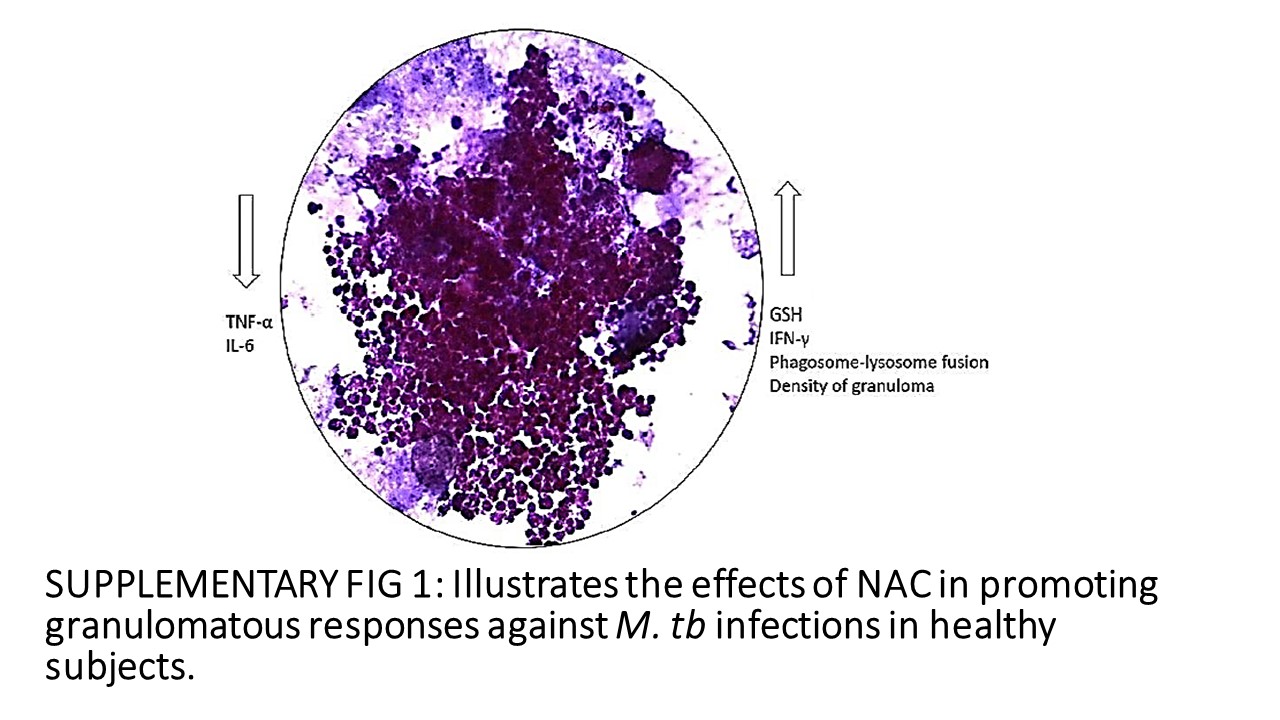

Supplement: Supplementary file 1 [file Image_1.JPEG]

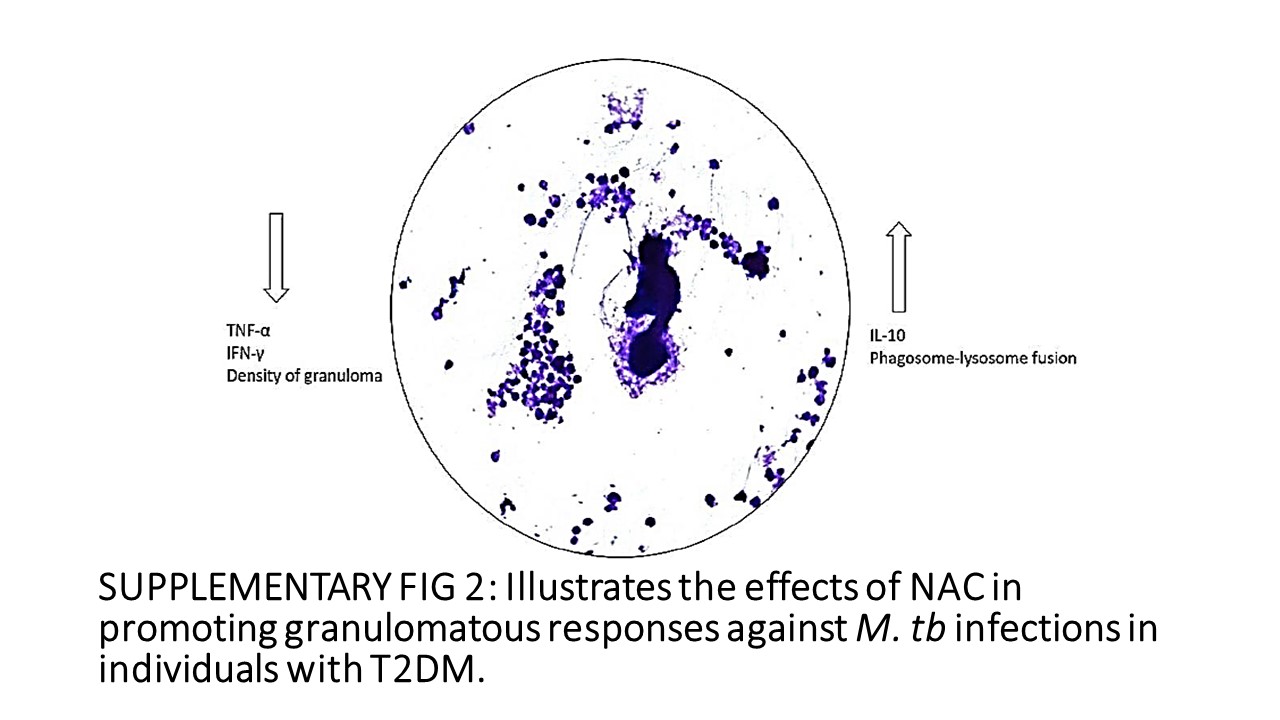

Supplement: Supplementary file 2 [file Image_2.JPEG]
